# In silico analysis of SARS-CoV-2 proteins as targets for clinically available drugs

**DOI:** 10.1038/s41598-022-08320-y

**Published:** 2022-03-29

**Authors:** Wallace K. B. Chan, Keith M. Olson, Jesse W. Wotring, Jonathan Z. Sexton, Heather A. Carlson, John R. Traynor

**Affiliations:** 1 Department of Pharmacology, University of Michigan, 2301 MSRBIII, 1150 W Medical Center Dr, Ann Arbor, MI 48190-5606 USA; 2 Edward F Domino Research Center, University of Michigan, Ann Arbor, MI 48190 USA; 3 Department of Medicinal Chemistry, University of Michigan, Ann Arbor, MI 48190 USA; 4 Department of Internal Medicine, University of Michigan, Ann Arbor, MI 48190 USA

**Keywords:** Target identification, Virtual drug screening

## Abstract

The ongoing pandemic caused by severe acute respiratory syndrome coronavirus 2 (SARS-CoV-2) requires treatments with rapid clinical translatability. Here we develop a multi-target and multi-ligand virtual screening method to identify FDA-approved drugs with potential activity against SARS-CoV-2 at traditional and understudied viral targets. 1,268 FDA-approved small molecule drugs were docked to 47 putative binding sites across 23 SARS-CoV-2 proteins. We compared drugs between binding sites and filtered out compounds that had no reported activity in an in vitro screen against SARS-CoV-2 infection of human liver (Huh-7) cells. This identified 17 “high-confidence”, and 97 “medium-confidence” drug-site pairs. The “high-confidence” group was subjected to molecular dynamics simulations to yield six compounds with stable binding poses at their optimal target proteins. Three drugs—amprenavir, levomefolic acid, and calcipotriol—were predicted to bind to 3 different sites on the spike protein, domperidone to the Mac1 domain of the non-structural protein (Nsp) 3, avanafil to Nsp15, and nintedanib to the nucleocapsid protein involved in packaging the viral RNA. Our “two-way” virtual docking screen also provides a framework to prioritize drugs for testing in future emergencies requiring rapidly available clinical drugs and/or treating diseases where a moderate number of targets are known.

## Introduction

The rapidly progressing coronavirus 2019 (COVID-19) pandemic, caused by the severe acute respiratory syndrome coronavirus 2 (SARS-CoV-2), requires therapeutic strategies that can quickly enter clinical trials to minimize the human, social, and economic impact. Repurposing of FDA-approved drugs has allowed potential anti-SARS-CoV-2 drugs to rapidly enter clinical trials—such as remdesivir, darunavir, and lopinavir^1–6^; repurposing efforts reduce drug development time, cost, and early failures due to safety concerns^7^. Unfortunately, most drug repurposing studies examine only a few classical viral targets/sites or use phenotypic screens that do not identify the drug target^8–11^. Pragmatically, drug repurposing success is limited by the number of clinically approved drugs and the number of SARS-CoV-2 targets for those drugs to act^12^. Moreover, most drugs repurposed for use against SARS-CoV-2 remain unproven in clinical trials, produce conflicting clinical results, or show marginal effectiveness^13–16^. Many people have now been vaccinated against the virus, but there are many who, for some reason or another, are not receiving vaccines and the possible scenario that variants of the virus may be resistant to current vaccines. Thus, there is an ongoing need for therapies, especially those acting at new or understudied SARS-CoV-2 proteins and sites.

The SARS-CoV-2 genome primarily consists of 4 structural proteins that form the virion and mediate cell entry and 16 nonstructural proteins (Nsps) that form the viral replication-transcription complex and inhibit host immune responses; both classes offer numerous potential antiviral targets. Notable structural proteins include the nucleocapsid (N), which packs viral RNA^17–19^, and the spike (S), a ~ 200 kDa homotrimeric protein that mediates viral entry through recognition by its receptor-binding domain (RBD) of the host angiotensin-converting enzyme 2 (ACE2) receptor^20^. After entry, the host ribosome synthesizes the precursor polyprotein, which the Nsp3 (or PLPro) and Nsp5 (or MPro) proteases activate via auto-cleavage into the 16 different Nsps^21^. Most drug repurposing studies screen the same classical antiviral targets, such as the active sites of Nsp3 or Nsp5, the spike RBD, or the RNA-dependent-polymerase active site (Nsp12) of the replication-transcription complex, using either in silico or experimental methods^16,20,22–24^.

Potential drugs for repurposing could target numerous additional protein sites. For example, drugs acting at allosteric sites or protein–protein interfaces (PPIs) on the spike protein might reduce viral entry to mammalian host cells^25^. Similarly, attractive targets include less studied domains/proteins, such as the Mac1 domain of Nsp3^26,27^ and the endonuclease Nsp15^28^, both likely involved in blocking the host immune response. Docking-based virtual screens enable the quick identification of leads by screening many drugs across single targets^26,27^, or single drugs across many targets^29^, or a combination of both^30^. For example, virtual screens have been used to identify plant products that may inhibit important viral proteins such as the main protease (Nsp 5 or Mpro)^31^, Nsp1^32^, RNA-dependent polymerase^33^, the spike protein binding domain^34^, Nsp15^33,35^ and Nsp16^36^. On the other hand, many virtual screens against SARS-CoV-2 targets are limited because they use single or relatively few sites, do not refine or prioritize drug/site poses, and/or ignore available biological data^37,38^. These factors prevent an objective comparison across different drugs and prioritize computational methods over biological data.

To address these drawbacks, we developed a "two-way" multi-ligand and multi-target virtual docking screen to study 1268 FDA-approved drugs at 48 established or predicted sites across 23 SARS-CoV-2 proteins to identify drug repurposing candidates for SARS-CoV-2 treatment. This strategy increases the probability of finding treatments by expanding the number of potential target sites and thus the effective library size. This approach also offers the possibility to develop drug combinations acting at two or more viral sites that could provide better anti-Covid-9 therapies compared to single target treatments^39^. We refined and normalized the docking scores into Z scores, and further, we compared our computational leads with experimental hits from a large phenotypic screen for anti-SARS-CoV-2 infection of human Huh-7 cells^10^. This resulted in the identification of 97 unique drug/site pairs across 45 sites on 23 SARS-CoV-2 proteins, from which we prioritized 17 compounds. We further assessed these hits for stability within their respective proposed binding sites using molecular dynamics and identified six potential repurposed drugs with direct antiviral potential against SARS-CoV-2.

## Results

### Identification of target proteins and binding sites

**Figure 1:**
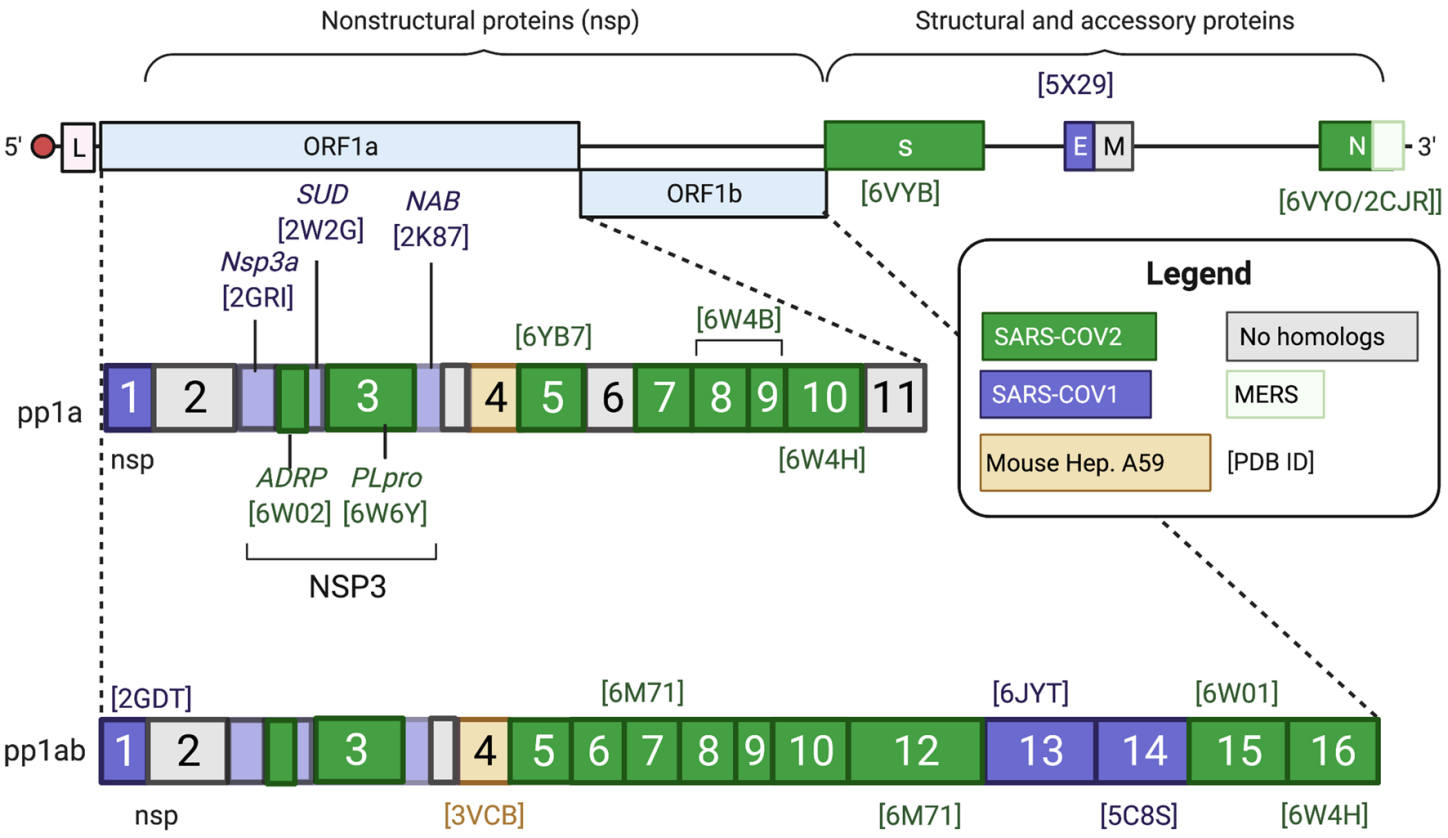
SARS-CoV-2 RNA Genome and Selected PDB Structures. Simplified SARS-CoV-2 RNA genome shows the proteins and PDB structures used for the *in-silico* drug repurposing screen. i. SARS-CoV-2 structures are in green. In the absence of a SARS-CoV-2 structure, homology models were built based on the closely related SARS-CoV-1 (purple), MERS (green outline), or Mouse Hepatitis A59 (yellow) [E value < 0.001]. Proteins with no SARS-CoV-2 or homologous structures were included in subsequent steps. The [PDB ID] of the chosen structure(s) is included above or below the appropriate gene. Created with Biorender.com.

Predicted SARS-CoV-2 protein sequences from the .NCBI.org database (Fig. 1) were subjected to a protein database (PDB) BLAST search to identify SARS-CoV-2 or related protein structures with an E value < 0.001. We selected SARS-CoV-2 protein/domain structures based on the lowest E value, then structural resolution if multiple SARS-CoV-2 structures existed (Fig. S1A). If no SARS-CoV-2 structures were available (as of May 2020), homology models were constructed based on solved structures from other coronaviruses, namely SARS-CoV-1, Middle East Respiratory Syndrome (MERS), or Mouse Hepatitis A59. Homology models were refined using the GB/VI force field model in Molecular Operating Environment (MOE)^40^, and the best scoring model for each protein was used in docking studies (Table S1). In all, this provided structures of 23 SARS-CoV-2 proteins, including 14 known structures and 9 homology models, for docking the repurposing library.

To virtually screen the repurposing library, we identified known and potential drug binding sites on each SARS-CoV-2 protein, including “classical” sites (e.g., spike protein RBD or protease active site), protein–protein interfaces (PPIs), sites with co-crystallized ligands, and putative allosteric sites. The "site finder" function in MOE was used to uncover additional potential binding sites on each protein based on their "Propensity for Ligand Binding (PLB)" indices, which scores binding pockets based on cavity size, depth, and amino-acid composition^41,42^. Sites that scored well with available crystal structures of SARS-CoV-2, SARS-CoV-1, MERS, or Mouse hepatitis A59 proteins were designated as PPIs. Other sites that scored well but had no apparent binding partner were designated as putative allosteric sites. Notably, this method identified most classical antiviral sites such as the active sites on Nsp3 and Nsp5 and the Nsp12 RNA binding sites, providing an internal validation of the approach; the only site the method did not identify was the spike protein recognition site for ACE2, most likely due to its flat and exposed surface. Lastly, sites with co-crystallized small molecules or nucleotides with an MW > 150 present were included as potential sites. In total, these analyses resulted in 48 binding sites (Table 1; see Table S2 for details of the amino-acid composition of each site).

**Table 1:** SARS-CoV-2 proteins, domains, and structure.

**Protein** *Domain*	**Species**	**E-value**	**PDB ID**	**Protein** *Domain*	**Species**	**E-value**	**PDB ID**
**NSP1**	SARS-CoV-1	E-52	2GDT	**NSP9**	SARS-CoV-2	–	6W4B
**NSP3**				**NSP10**	SARS-CoV-2	E-79	6W4H
*NSP3a (834–930)*	SARS-CoV-1	E-24	2GRI	**NSP12/RdRp**	SARS-CoV-2	–	6M71
*ADRP/MAC-1 (1001–1183)*	SARS-CoV-2	0	6W6Y	**NSP13**	SARS-CoV-1	E-54	6JYT
*SUD (1184–1540)*	SARS-CoV-1	E-105	2W2G	**NSP14**	SARS-CoV-1	E-33	5C8S
*PLPro (1640–1887)*	SARS-CoV-2	E-6	6W6Y	**NSP15**	SARS-CoV-2	–	6W01
*NAB (1888–1997)*	SARS-CoV-1	E-41	2K87	**NSP16**	SARS-CoV-2	–	6W4H
**NSP 4**	Mouse Hep. A59	E-24	3VCB	**S**—**Spike**	SARS-CoV-2	–	6VYB (open), 6VXX (closed), 6VW1 (w/ hACE2)
**NSP5**	SARS-CoV-2	–	6YB7	**E Protein**	SARS-CoV-1	E-9	5X29
**NSP7**	SARS-CoV-2	E-25	6M71	**Nucleoprotein**	SARS-CoV-2	0	6VYO
**NSP8**	SARS-CoV-2	E-25	6M71	*CTD (248-265)*	MERS	E-59	2CJR

### Docking-based “two-way” virtual screening campaign

**Figure 2:**
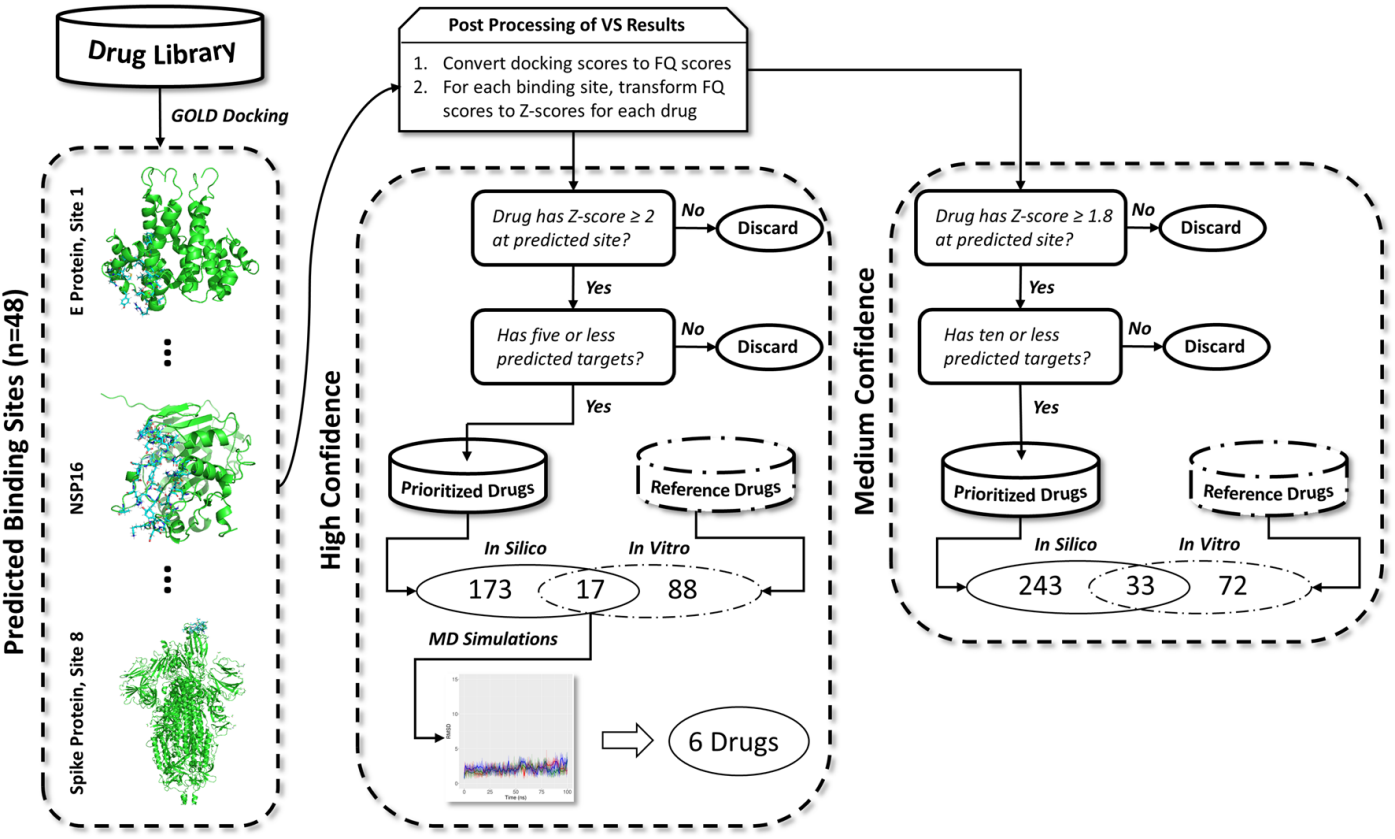
Pipeline for docking-based, “two-way” virtual screening strategy. GOLD software was used to dock the drug library against each of the 48 predicted binding sites. All docking scores were converted to fit quality (FQ) scores. Subsequently, the FQ scores of each drug were transformed into Z scores for each site. Out of the 1,268 FDA-approved drugs, 190 were prioritized with a Z score ≥ 2 at 5 or fewer target sites; these were considered high confidence hits. Consequently, 17 overlapped with a set of drugs previously known to have marginal to high potency against SARS-CoV-2 in vitro and were considered virtual hits^10^. These were then subjected to MD simulations, in which 6 drugs were observed to bind stably to their respective target protein. A medium confidence set of drugs was also prepared based on a Z score ≥ 1.8, which prioritized 276 drugs; 33 of these overlapped with the reference drugs. Overall, there were 34 and 97 unique drug-site pairs for the high- and medium confidence hits respectively that overlapped with the reference drugs. In-silico, prioritized drugs are shown in cylinders with solid lines and reference drugs with in vitro activity against SARS-CoV-2 in cylinders with dashed-dotted lines. Prepared using PowerPoint Presentation Software.

The pipeline used for our virtual screening campaign is illustrated in Fig. 2. Using GOLD software (Cambridge Crystallographic Data Center, see methods), we docked 1268 FDA-approved small molecules from DrugBank (see methods) to all 48 binding sites across 23 SARS-CoV-2 proteins. Docking scores were normalized to fit quality (FQ) scores to minimize bias towards higher molecular weight compounds and rescue low MW drugs that would otherwise have been discarded (Fig. S3)**.** Subsequently, FQ scores were transformed into Z scores (Fig. S2) for each drug at each binding site to directly compare across drug-site pairs. Both “high confidence” as defined by a Z score of 2 or greater and “medium confidence” as defined by a Z score of 1.8 or greater strategies were employed to identify compounds. Of the drugs that had Z ≥ 2 at any given site, a decreasing trend was observed in the number of predicted sites bound with respect to frequency of drugs; 5 sites were chosen as the cutoff point. Additionally, all 48 predicted sites from the SARS-CoV-2 proteins bound at least one predicted drug with Z ≥ 2, and these drugs appeared relatively evenly distributed among the sites. From this, we characterized 190 drugs (15% of the library) as high confidence hits. By relaxing the screening criteria to Z ≥ 1.8 and limiting the number of interacting sites for any one drug to 10 different sites, we identified an additional 86 compounds for a total of 276 hits (22% of the virtual library). These medium confidence compounds were predicted to interact with various proteins and binding sites yielding 97 unique drug-binding site pairs across 45 different sites on the 23 SARS-CoV-2 proteins investigated (Table S3). The criteria chosen for the high confidence hits allowed us to select a reasonably sized pool of drugs while minimizing promiscuity. We loosened the cut-offs to identify medium confidence hits that would allow for more false negatives to enter the hit pool, while limiting the number of false positives; further lowering of the criteria would have caused the inclusion of many false positives This is illustrated graphically in Fig. S2.

### Comparison of in silico hits with experimental data

**Figure 3:**
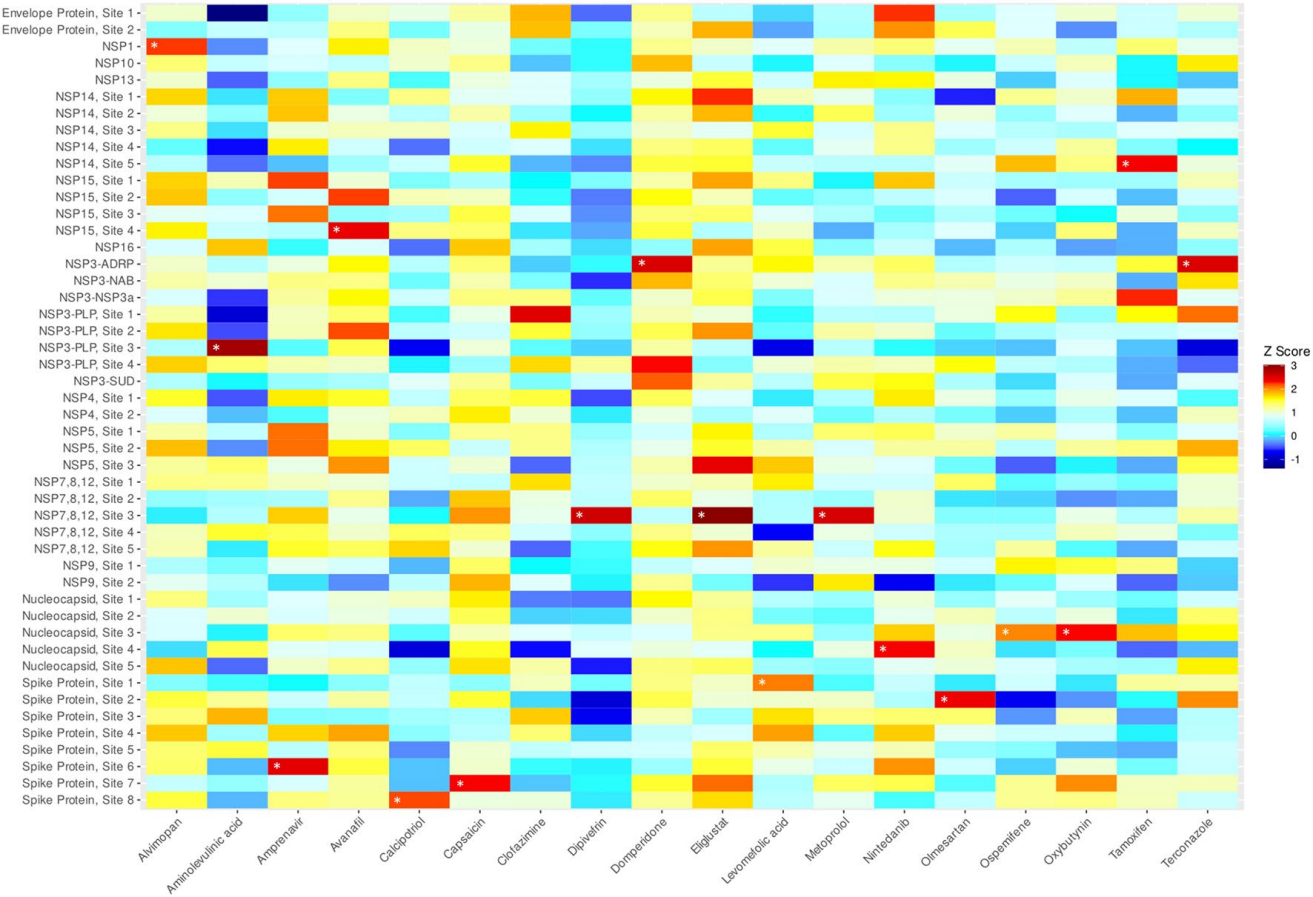
Heat map of hits from composite virtual screening campaign. Under our prioritization scheme, 17 high confidence, FDA-approved drugs were predicted to interact in 34 unique drug-site combinations. White asterisks denote the systems selected for molecular dynamics simulations. The heat map was generated with R language using the ggplot2 package.

A recent report of drug repurposing identified 15 drugs in our library as having high (20–765 nM) potency at reducing SARS-CoV-2 infection in human Huh-7 cells^10^. Only one of these compounds, domperidone, was identified in our virtual screen using the high confidence criteria. However, the experimental study identified an additional 88 hits that were effective at 2 μM or better that have not yet been further characterized (Table S4). Of these compounds, 17, including domperidone, overlapped with the high confidence group of drugs. Our analysis predicted these 17 drugs to bind 25 different sites with 33 different drug-site combinations (Table S5). A heat map of these 17 compounds at each of the 48 predicted or actual SARS-CoV-2 protein binding sites is shown in Fig. 3. Of the hits, 8 had Z values ≥ 2 at more than one site, for example, amprenavir at Nsp15 and Nsp5, and avanafil at two distinct sites on Nsp15. Overall, 72 of the high priority drugs identified by docking did not map with the experimentally determined hits, and 87 of the experimental hits did not appear in our high confidence list (Fig. 3). When the medium confidence hits were included, a further 16 drugs were identified as active in both the in silico and experimental screen (Table S3). Indeed, five of these drugs were potent inhibitors of SARS-CoV-2 infection of Huh-7 cells^10a,b^, namely Bosutinib, IC_50_ = 20 nM; Fedratinib, IC_50_ = 24 nM; domperidone, IC_50 =_ 44 nM; remdesivir, IC_50_ = 97 nM; amiodarone, IC_50_ = 167 nM; lomitapide, IC_50_ = 765 nM).

### Molecular dynamics simulations and trajectory analysis

To account for the limitations of molecular docking, which include the absence of solvent, minimal sidechain and backbone flexibility, we performed molecular dynamics simulations on our 17 top in-silico hits using the drug-binding site pairing that gave the highest Z score (Table S5) to evaluate the stability of the bound drugs in a quasi-physiological environment. Simulations were run for 100 ns in three independent trials followed by a molecular dynamics trajectory analysis to assess protein and drug stability. From these simulations, we further refined our list down to 6 drugs that met the following criteria: (1) the final snapshot shows the same drug pose in at least 2 replicates, (2) the drug RMSD converges for at least two of the replicates, and (3) the protein remains stable during the simulation. 6 drug/site pairs met these criteria. (Figs. S4 and S5). Drug RMSDs from those systems that did not meet these criteria are shown in Fig. S6.

Most proteins remained stable throughout the simulation (Fig. S4). Nsp14 moderately diverged from the original structure, likely because we used a homology model. In addition, in the presence of ospemifene, the nucleocapsid complex partially or entirely destabilized in all three replicates (Fig. S4), although this did not occur in the presence of nintedanib or oxybutynin. Lastly, the spike protein showed a moderate shift in all tests; we ascribe this to the numerous loops in the structure, which are not observed in the X-ray analysis and had to be modeled.

**Figure 4:**
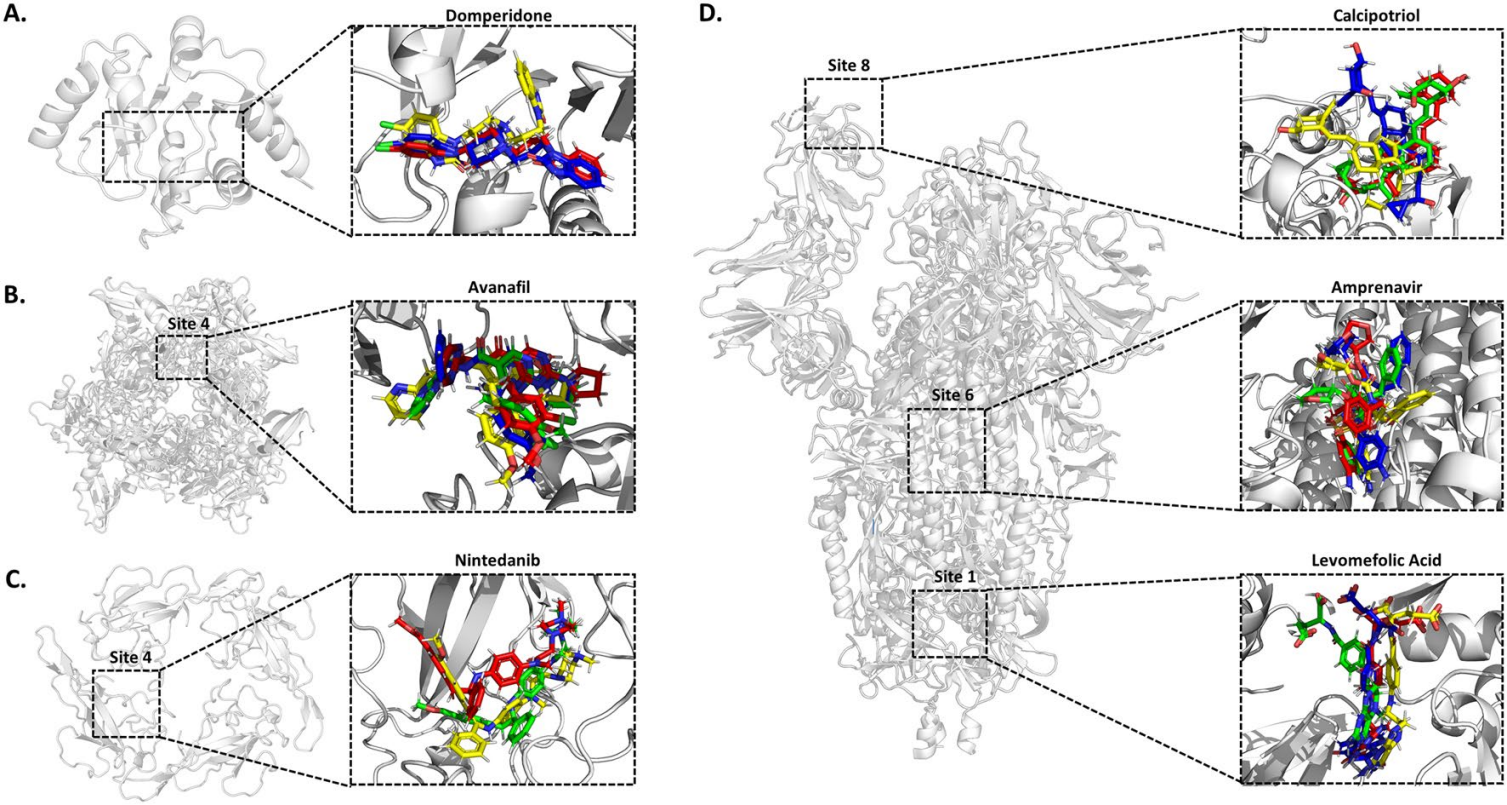
Final snapshots from molecular dynamics simulations for stable drug-site systems. The docked drug pose is shown in yellow, while the final pose after three independent 100 ns molecular dynamics simulations is shown in red (1), green (2), and blue (3). (**A**) ADRP domain of NSP3 with domperidone docking and final molecular dynamic simulation poses. (**B**) Site 4 of NSP15 with Avanafil docking and final molecular dynamic simulation poses shown. ADP is shown in dark red. (**C**) Site 4 of the RNA-binding domain of the nucleocapsid protein with nintedanib docking and final molecular dynamic simulation poses shown. (**D**) Spike protein sites 1, 6, and 8 with levomefolic acid, amprenavir, and calcipotriol docking and final molecular dynamic simulation pose shown. In one trial, domperidone and nintedanib migrated to another part of the protein (not shown). Please see Figs. S7–S12 for additional descriptions of the trajectories. Molecular graphics were generated using PyMOL and the figure was prepared using PowerPoint Presentation Software.

In contrast to the stability of the proteins, only six drugs (domperidone, avanafil, nintedanib, levomefolic acid, amprenavir, and calcipotriol) remained in their predicted binding pockets throughout the simulations (Fig. S5). Most drugs either migrated to another site or diffused into the solvent early in the simulation, including aminolevulinic acid and metoprolol, or showed instability in two or more replicates. With terconazole, the ligand trajectories converged in two trials, achieving a similar ligand orientation but outside the predicted site. With alvimopan and dipivefrin, the drugs fully dissociated from the protein in one replicate, migrated to another site in another, and remained stable in yet another. Overall, the study predicted that: domperidone and avanafil bound stably with different Nsps, that levomefolic acid, amprenavir, and calcipotriol bound to the spike protein, but at three different sites, and that nintedanib bound stably with the nucleocapsid (Fig. 4).

Domperidone bound to the ADP-ribose-1-monophosphatase (ADRP or Mac1) active site of Nsp3^41-44^  (Fig. S7A). In two of three simulations, a cryptic pocket opened to accommodate the 1,3-benzodiazol-2-one moiety of the drug (Moiety 1B) (Fig. S7B/C). This small cavity was formed by Leu160 moving outward and Phe156 rotating inward to stabilize drug binding with a side-to-face aromatic interaction (Fig. S7E). In the presence of domperidone, the binding pocket adopted a more open conformation in the 'clamp' region at the ADP ribose site, by an outward movement of Ile131 (Fig. S7D), perhaps due to relaxation from the crystal structure. The avanafil pose was at the interface between Nsp15 homomers (Figs. 4, and S8A/B), buried in a hydrophilic pocket with access to a solvent channel that leads to the protein surface (Fig. S8C). While the simulations with avanafil employed a single site, the enzymatically active form of Nsp15 is a homohexamer such that avanafil could bind at any of the six comparable interfaces^28^ to disrupt the quaternary structure. However, our MD timescale was not long enough to predict complete protein disassociation.

The spike protein is an obvious target for antiviral activity. Levomefolic acid showed binding to a cavity between the spike protein monomers in the trimeric state (Fig. S9). Amprenavir exhibited modest conformational flexibility within a putative hydrophobic pocket on the spike protein subunit 2, located near the S2' cleavage site between residues 815/816^45^ (Fig. S10). In this pocket, the sulfonamide-containing moiety remained stable and buried in the pocket, although the benzyl and oxolan-3-yl acetyl groups were more dynamic (Fig. S10B/C). The drug also creates several contacts with the fusion peptide^46^ (Fig. S10A). Calcipotriol interacted with the RBD. Calcipotriol was positioned flat against the protein, though simulations revealed that the (1R,3S)-4-methylidenecyclohexane -1,3-diol-containing portion of the drug was mobile and preferred to be in the solution phase (Fig. S11A/C), while the rest of the drug was firmly sequestered within a hydrophobic pocket (Fig. S11B/C). This resulted in calcipotriol partially blocking the ACE2 receptor binding site.

Finally, nintedanib was observed to bind stably to the RNA-binding pore of the nucleocapsid (Fig. S12). As the nucleocapsid is a homotetramer, it could be predicted to bind 4 molecules of nintedanib.

## Discussion

**Figure 5:**
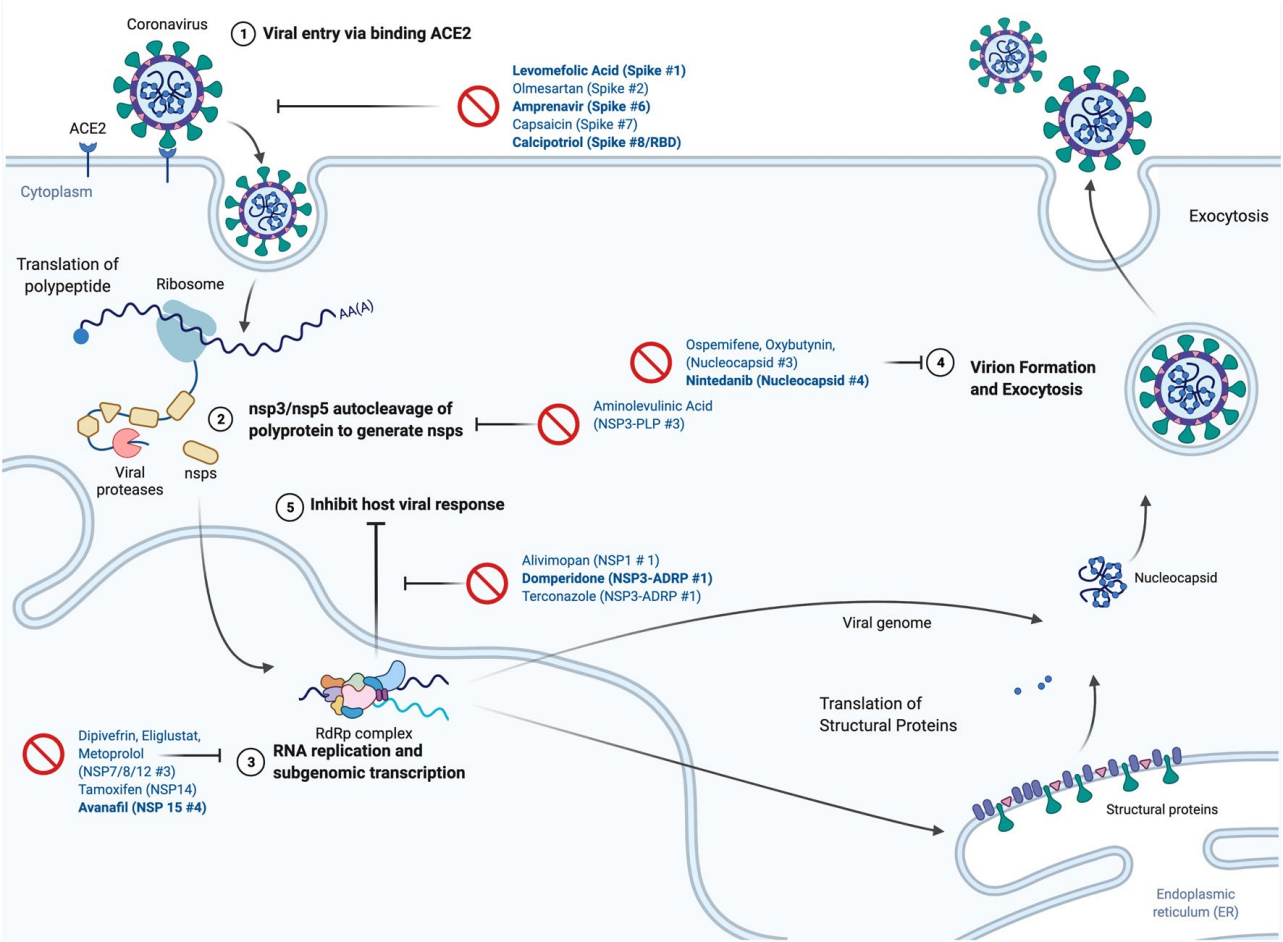
Predicted Sites and Mechanism of 17 “High-Confidence” drug/site pairs. The 17 drugs with Z score ≥ 2 at 5 or fewer target sites and previously identified anti-SARS-CoV-2 activity in vitro*.* The predicted drug/site pairs and commonly drugged steps in the (simplified) viral life cycle . including (1) blockade of viral entry mediated via the S protein recognizing the host ACE2; (2) inhibition of viral proteases NSP3 and NSP5 which auto-cleave and activate the 16 nsps, (3) inhibition of the viral replication/transcription complex; (4) blockade of virion formation, RNA packaging, and exocytosis, and (5) blockade of viral proteins that attenuate the host immune response. Drugs names in bold indicate are “High-Confidence” hits predicted to form stable drug/receptor interaction in molecular dynamics simulations, non-bolded drugs failed to form stable complexes in the MD simulations. Drug lists ordered as, “Drug (Protein Site #)”. Created with biorender.com.

To identify potential new drug repurposing candidates with anti-SARS-CoV-2 activity, we designed a "two-way" virtual docking screen of FDA-approved drugs at 48 diverse sites, including classic antiviral sites, PPIs, sites with co-crystallized small molecules, and predicted allosteric sites to maximize the chance of success. To compare drug/site pairs, we report a prioritization scheme that normalized all docking scores to Z scores^38^. To the best of our knowledge, this is the first application of such a methodology to a virtual screening pipeline and allowed us to identify a diverse selection of potential anti-SARs-CoV-2 drugs. This resulted in the in silico characterization of many unique drug-site pairs while retaining information from 48 single-site screens. Although we do not yet have experimental evidence that all of these sites, particularly the ones that are not classical targets for antiviral drugs, are able to modulate viral protein activity we did identify 15 compounds from our screen that have anti-SARS-CoV-2 activity in an in vitro screen in human Huh-7 cells (Fig. 5), and 6 of these showed stable interactions with SAR-CoV-2 proteins in molecular dynamics simulations (Fig. 5). Unfortunately, domperidone, which was highly potent in the experimental screen and a high confidence hit in the in silico screen, was inactive against the virus in a follow-up study that examined anti-viral activity in four other cell lines^10^, which may suggest interactions with mammalian cell targets rather than viral targets, or that binding at the Mac3 site of NSP3 does not produce viral inhibition. This host cell target is unlikely to be the dopamine D2 receptor, the clinical target for domperidone in treating nausea and vomiting, since there is no evidence that the D2 receptors are expressed in Huh-7 cells; this suggests a novel, but cell-specific, mechanism of antiviral action^47^. Nonetheless, our approach does provide a framework for the in silico screening of repurposed drug libraries against many protein targets and a starting point to develop novel antiviral drugs from leads predicted in this study.

Previous virtual drug repurposing screens against multiple SARS-CoV-2 targets suffer from several limitations, including the use of relatively few sites and/or too many sites obtained from automated methods, reliance on docking scores alone to prioritize drugs that favorably bias large molecular weight ligands, biased comparison of drugs between binding sites, the absence of molecular dynamics simulations to further prioritize lead docked structures^37,38^, and a lack of use of known biological data on anti-SARS-CoV-2^29,30^ activity. Since many of the putative sites and drug-target interactions we have identified are novel we were unable to retrospectively benchmark our methodology. However, an original aspect of the present study, in comparison to previous in silico work, is that we employed biological data in our decision tree. These data were obtained from a phenotypic cell-based anti-viral assay. Such a methodology is expected to discover drugs that bind/modulate both viral and/or host factors. The cross-over hit rate between our virtual screen and the phenotypic screen was not high, but this is most likely because the in silico methodology only identified the subset of compounds acting at viral proteins and not on host factors. Additionally, binding sites among the proteins analyzed may not have been pre-formed in the crystal structures. For example, a spike protein crystal structure shows linoleic acid bound in a novel pocket not previously observed in other crystal structures^48^.

A second novel aspect of our study was the conversion of docking scores to fit quality (FQ) scores, which is essentially a scaled ligand efficiency metric that has not previously been reported in virtual screening campaigns. Additionally, we normalized FQ scores to Z scores to prioritize different drug-site pairs, a method that had previously only been employed to analyze adverse drug reactions^38^. From the Z-scores, we identified a group of potential anti-SARS-CoV-2 drugs. In traditional virtual screening methods, the goal is to minimize false positives^49^, but since the size of the FDA-approved drug library is fixed, we included 97 “medium-confidence” drug/site pairs using less-stringent criteria. These 97 drug/site pairs represent 0.002% of the possible 60,864 combinations (48 sites × 1268 drugs). This provides a resource for further investigating potential SARS-CoV-2 drugs identified in phenotypic screens without an obvious target, where target deconvolution remains a major barrier^50^. Moreover, several of the identified drugs are predicted to act at multiple sites across the same or different proteins, suggesting drug combinations may produce additive or even synergistic antiviral activity^29,30^.

One caveat to our work is that we do not know if occupancy of many of putative binding sites we identified will lead to modulation of viral protein activity and it will be important in the future to substantiate the role of each of the putative sites in the inhibition of the target. Such a large study is outside the scope of this study. Nonetheless, Table S2 lists all the sites by their type, whether classical (i.e., well-validated), putative allosteric (i.e., not necessarily validated but predicted site without an established mechanism), or protein–protein interactions. Inclusion of this information will provide a starting point for future studies, especially to help study newly discovered anti-SARS-CoV-2 compounds that may not have a known mechanism of action.

Overall, we identified 17 compounds in the high confidence group that overlapped with compounds that showed some degree of antiviral activity against SARS-CoV-2 infection in Huh-7 cells. These compounds bound to 48 sites on the virus proteins. The highest scoring drug-binding site interactions were subjected to molecular dynamics to provide six stable drug-binding site pairs. Moreover, the predicted binding site for each drug provides a logical rationale as to why the compounds could interfere with the activities of their target proteins and so possess antiviral activity. Domperidone bound to the ADP-ribose site on the Mac1 domain of Nsp3. This would compete with the hydrolysis of the natural substrate, which is associated with virulence and the ability to evade host immune response^43,44^. In fact, preclinical models of SARS-CoV-2, SARS-CoV-1, and MERS-CoV^27^ suggest Mac1 inhibitors may act as broad-spectrum antivirals. Avanafil, a phosphodiesterase type 5 inhibitor bound to a putative site at the interior of the Nsp15 hexamer complex. This complex is an uridylate-specific endoribonuclease that processes viral RNA to avoid activation of host double-stranded RNA (dsRNA) sensors^51^. Active sites on the enzyme are located externally^28^, so if it were active, avanafil would have to allosterically modulate Nsp15 activity, although some evidence indicates Nsp15 can jeopardize the immune response independent of NendoU activity^50,52^. Nintedanib is a tyrosine kinase inhibitor bound to the nucleocapsid, a protein essential for packaging RNA during viral assembly, replication, and RNA transcription. It is also worth noting that ospemifene, a selective estrogen receptor modulator, was identified as binding to the nucleocapsid protein. This was not included in the final choice of compounds since molecular dynamics simulations of the binding site suggested drug binding decreased the protein’s stability. On the other hand, this effect may be an important property because recent reports show that destabilization of the native oligomers by stabilizing non-native oligomers of the N-terminal domain with small molecules, produced antiviral activity^53^.

The spike protein is a principal target for COVID-19 therapeutics due to its essential role in viral entry into the host cell. Amprenavir, levomefolic acid, and calcipotriol were predicted to interact with the spike protein at different locations: a putative allosteric site, a PPI, and the RBD, respectively. Amprenavir bound stably to a potential allosteric hydrophobic pocket on the spike protein subunit 2, which the host TMPMRSS2 protease cleaves to enable viral entry, forming predicted contacts with the fusion peptide^44,46^. This acts as a grappling hook, moving into the host membrane and mediating fusion. Levomefolic acid, by binding to a cavity between spike protein oligomer interface, could function as a PPI to prevent the formation of the active heterotrimer. Calcipotriol was predicted to interact stably with the RBD of the spike protein and could ostensibly interfere with the recognition of the host ACE2 receptor. It is also notable that amprenavir was a medium confidence hit at Nsp5 (also known as main protease, MPro, or 3CLPro), which fits with its use as an HIV protease inhibitor, providing two potential mechanisms for this drug.

Unfortunately, although shown to have activity in Huh-7 cells including domperidone which was particularly potent, none of our top six repurposed candidates showed activity against SARS-CoV-2 infection in follow-up experiments using a variety of cell lines^10^, although, in addition to Huh-7 cells, amprenavir has been reported to have moderate anti-SARS-CoV-2 activity against infected Vero E6 cells^52,54^. This could mean the compounds are not effective against the degree of viral loads and /or ease of viral infection in all cell lines or, and more likely, the drugs were acting on host factors in the Huh-7 cells. In support of this, an experimental comparison of re-purposed drugs across five different human-derived cell lines showed domperidone to be only active against SARS-CoV-2 infection in Huh-7 cells but not in Vero-E6 cells, Caco-2 E6 cells, or iAEC2 cells^10^. Drugs exerting efficacy through direct interactions with viral proteins should be less dependent on the host cell system, and especially entry inhibitors that act in the extracellular environment. Remdesivir, the preclinical drug Z-FA-FMK, an irreversible inhibitor of cysteine proteases, amiodarone, ipratropium bromide, lomitapide, and lactoferrin, were active across multiple cell lines^10^ indicating a stronger potential towards direct viral binding. Remdesivir was identified as a medium confidence, not high confidence hit in our in silico screen, presumably because it is a pro-drug and requires conversion to its active metabolite (GS-441524 triphosphate) to inhibit viral RNA polymerase.

A recent meta-analysis of FDA-approved drugs for experimental anti-SARS-CoV-2 activity listed none of our final six candidates, although calcipotriene, like calcipotriol, a synthetic vitamin D analog, is listed^55^. Notably, vitamin D deficiency corresponds to a greater risk of testing positive for COVID-19^56,57^. Nonetheless, it is feasible that drug combinations could inhibit several viral targets, for example, domperidone at Nsp3 together with avanafil at Nsp15, or even a single viral target in different ways, for instance, amprenavir, levomefolic acid, and calcipotriol at the spike protein could provide sufficient antiviral efficacy.

Independently, recent publications on SARS-CoV-2 have confirmed several of our predicted drug sites, for example allosteric sites at the Spike protein^58^, validating our approach. Furthermore, the present study provides a resource to aid the deconvolution of interactions of viral protein sites from phenotypic screens when the actual target is unknown. Moreover, this method and our results can assist in scaffold identification for further development using structure–activity-relationship studies to identify novel drugs against a wide variety of potential targets in SARS-CoV-2. Finally, the “two-way” virtual docking screen we implemented supplies a framework for future emergencies requiring rapidly available clinical drugs for treating diseases where a moderate number of targets are known.

## Methods

### Protein structures and homology models

The SARS-CoV-2 predicted protein sequences were obtained from the NCBI.org database. The putative protein sequences for each protein were then analyzed using a BLAST to determine sequences with an E score < 0.001. If a SARS-CoV-2 structure was available, that was prioritized. If multiple SARS-CoV-2 structures were available, the highest resolution structure was chosen (Fig. S1). A homology model was built in cases where different domains were available from SARS-CoV-1, MERS, or Mouse Hepatitis A59. SARS-CoV-2 sequences from NCBI were aligned with template structure sequences (Table S1). A homology model using MOE (Molecular Operating Environment)^40^ was built for each domain or protein and scored via the GBVI/WSA dG (Generalized-Born Volume Integral/Weighted Surface area) force field function in MOE with a refinement value of 0.5 kcal/mol. Refinements were applied to both intermediate and final models. For each protein, 15 different structures were generated with two different sidechain orientations for a total of 30 models each, using the default settings in MOE. The best scoring model was inspected using the “protein geometry” stereochemical quality evaluation tools in MOE (Table S1).

### Site identification and protein preparation

All structures were prepared using the MOE ''Quick Prep” to determine protonation state, calculate partial charges, and relax strain. Using the structures indicated in Tables 1 and S2^28,59–77,79,80^, sites were chosen using computational and structural analysis (Fig. S1). First, the MOE site finder function was used to identify potential sites. Sites were selected if there was a PLB (Propensity for Ligand Binding) index of > 3 or > 1.5 if no other sites were identified. Based on manual inspection of the crystal structures, putative catalytic, nucleotide-binding, or drug binding sites were included, if not otherwise incorporated. Next protein–protein interaction sites were chosen based on co-crystallized SARS-CoV-2 protein complexes. Lastly, SARS-CoV-2 structures were overlaid with homologous structures to identify sites of co-crystallized ligands in the homologous structures. In cases of oligomers, only non-redundant sites were selected. Lastly, if solved structures contained small molecules (MW > 150 Da), their position on the protein was considered a potential site.

### Construction of virtual drug library

Drug structures were obtained from DrugBank (version 5.1.5, 2020-01-0’ release, ‘3D structures.sdf’)^81^ and imported into a Molecular Operating Environment (MOE, version 2019.0101) database. The pipeline for constructing our virtual drug library is shown in Fig. S2A. Drugs under the label 'approved' corresponding to FDA-approved drugs were isolated from the full set. Afterward, chemical structures were subjected to the 'wash' protocol in MOE to remove salts, generate 3D conformations, and protonated based on the predicted dominant species at pH 7.4. Lastly, a conformational search using LowModeMD with default settings predicted the lowest-energy conformer for each drug. Our final virtual library consisted of 1268 compounds based on the following criteria: (1) presence in DrugBank, (2) not a protein or peptide, (3) molecular weight between 100 and 700 Da, and 4) contain fewer than 15 rotatable bonds.

### Docking-based, “two-way” virtual screen

GOLD (version 5.8.1; Cambridge Crystallographic Data Center or CCDC) was used for molecular docking of the virtual library against the 47 predicted binding sites at 21 SARS-CoV-2 proteins or domains (Table 1)^82^. From the MOE Site Finder predictions, residues within 4.5 Å of the alpha spheres were used to define the cavity. For each drug, 50 independent genetic algorithm runs were performed using the highest conformational search efficiency setting ('very flexible') and the default diverse solutions options. Ring conformations were explored using the 'flip ring conformers’ and 'match conformations' options. Poses were evaluated with the ChemPLP scoring function, and the top-scoring pose of each drug at each site was kept for further analysis.

Since the drugs in our virtual library span between 100 and 700 Da, we normalized the docking scores based on their molecular weight by converting them into ligand efficiency (LE) values as previously reported^83^:

1 $$LE = \frac{Docking\,\, Score}{{HA}}$$

where HA is the number of heavy atoms. For similar reasons, a scaling calculation was used to account^83,84^:

2 $$LE\_Scale = 0.0715 + \frac{7.5328}{{HA}} + \frac{25.7079}{{HA^{2} }} - \frac{361.4722}{{HA^{3} }}$$

LE and LE Scales were used to calculate a fit quality (FQ) score:

3 $$FQ = \frac{LE}{{LE\_scale}}$$

To make each drug comparable between binding sites, their FQ scores were converted into Z-scores:

4 $$Z = \frac{FQ - \mu }{\sigma }$$

where the FQ is the FQ score of a drug at a given site. μ and σ are the mean and standard deviation, respectively, of the FQ scores at the specified site, which were normally distributed at each site. Ultimately, the poses of drugs with Z ≥ 2 at five binding sites or less were retained and considered virtual hits. Drugs that scored well with numerous targets were not considered due to the greater possibility of being promiscuous binders. Custom Perl and R scripts were used to parse, analyze, and visualize the virtual screening results.

### Pose refinement of experimentally verified virtual hits

The 17 drugs that matched experimental observations and possessed docked poses scoring Z ≥ 2 at five binding sites or less were subjected to pose refinement in MOE using the 'Induced Fit' option. Sidechains could freely rotate with a maximum of 10,000 iterations and scored using GBVI/WSA to implicitly account for solvation. Default parameters were used for the other settings.

### Molecular dynamics simulations

All protein structures were prepared with MOE (version 2019.0101)^38,40^ for molecular dynamics simulations. N-linked glycans were removed from the spike protein. N- and C-termini were capped with acetate and N-methyl amide, respectively. Missing loops were modeled with the “Loop Modeler' module. Hydrogen atoms were added, and the protonation state of all relevant residues was assigned at pH 7.0. Prepared structures were saved as PDB files that conformed to AMBER atom typing. Ligands were parameterized with Antechamber using the GAFF2 forcefield^85^, where charges were assigned using the AM1-BCC method. From AmberTools18, tleap was utilized to prepare the input files using the prepared structure with the AMBER ff14SB force field^86^, while disulfide bonds were manually assigned where pertinent. Each system was padded with 15 Å of TIP3P water on all sides and neutralized with an apparent number of either Na + or Cl- ions (Table S6).

The GPU implementation of PMEMD from AMBER18 was employed for conventional molecular dynamics simulations^87^. An initial round of minimization was performed on the systems with a 10 kcal/mol·Å^2^ harmonic restraint on the protein, which consisted of 2500 steps of steepest descent followed by 2500 steps of the conjugate gradient. The minimization was repeated a second time but without introducing the harmonic restraint. Subsequently, the systems were gradually heated in the NVT ensemble from 0 to 100 K for 12.5 ps, then from 100 to 310.15 K in the NPT ensemble for 125 ps at 1 bar; a 1 fs time step was used, along with a 10 kcal/mol·Å^2^ harmonic restraint on the protein and ligand. Upon reaching the target temperature, the systems were equilibrated in the NPT ensemble at 310.15 K and 1 bar with a 2 fs time step. Starting with a 5 kcal/mol·Å^2^ harmonic restraint on the protein and ligand, the restraint was diminished by 1 kcal/mol·Å^2^ every 500 ps for a total of 2.5 ns, then by 0.1 kcal/mol·Å^2^ every 500 ps for a total of 5 ns. Production simulations were run at 310.15 K (37 °C for comparison with biological studies) and 1 bar in the NPT ensemble for 100 ns with a 2 fs time step. All bond lengths involving hydrogen were constrained with the SHAKE algorithm. Particle Mesh Ewald summation was used for long-range electrostatics, while non-bonded interactions were cut off at 9.0 Å. Periodic boundary conditions were applied to all heating, equilibration, and production runs. The Langevin thermostat and Monte Carlo barostat were used where applicable. Three independent production runs were carried out for each of the systems using randomized velocities. Individual runs were performed on a single Nvidia GeForce RTX 2080 Ti GPU on a local computer cluster.

Custom CPPTRAJ scripts were used to process all trajectories of production simulations. All components were imaged to wrap all contents back to the periodic cell, and all waters and ions were stripped for analysis. Structure alignments were performed in reference to the docked proteins using the backbone atoms (C, Cα, N). Root mean square deviations of both the proteins and drugs were calculated following protein structure alignment.

### Data visualization and diagram construction

Diagrams were created in Microsoft PowerPoint. Graphs were generated using the R statistical programming language with the open-source data visualization package ggplot2 (version 3.3.3). Molecular visualization images for protein–ligand complexes were produced using PyMOL 2.5 (Schrodinger) or MOE version 2019.0101.

## Supplementary Information

Supplementary Information.
