# Supplementary material for: In silico analysis of SARS-CoV-2 proteins as targets for clinically available drugs
Source: Sci Rep. 2022 Mar 29;12:5320. doi: 10.1038/s41598-022-08320-y (PMC8963407; doi:10.1038/s41598-022-08320-y)
Supplement: Supplementary file 1 — Supplementary Information. [file 41598_2022_8320_MOESM1_ESM.pdf]

SUPPORTING INFORMATION

In silico analysis of SARS-CoV-2 proteins as targets for clinically available drugs

Wallace K.B. Chan[1,2*], Keith M. Olson[1,2*], Jesse W. Wotring[3], Jonathan Z. Sexton[3,4], Heather A. Carlson [3], John R. Traynor[1,2,3,†]

[1]Department of Pharmacology, [2] Edward F Domino Research Center, [3]Department of Medicinal Chemistry, [4]Department of Internal Medicine, University of Michigan, Ann Arbor, MI 48190, USA

Table of contents

1. **Supporting Figures**

- **Figure S1**: Flowchart for preparation of SARS-CoV-2 protein structures
- **Figure S2**: Z score distributions of drugs for each predicted site
- **Figure S3**: Molecular weight dependence of scoring methods
- **Figure S4**: Protein RMSDs from molecular dynamics simulations trajectories
- **Figure S5**: Drug RMSDs from trajectories of successful systems
- **Figure S6**: Drug RMSDs from trajectories of failed systems
- **Figure S7**: Domperidone is predicted to displace ADP ribose from the ADRP domain of NSP3
- **Figure S8**: Avanafil interacts with an allosteric site on NSP15
- **Figure S9**: Levomefolic acid interacts with a putative allosteric site formed between the trimeric spike protein complex
- **Figure S10**: Amprenavir interacts stably with a putative allosteric site on the spike protein
- **Figure S11**: Calcipotriol maintains a stable interaction with the RBD of the spike protein
- **Figure S12.** Nintedanib interacts with the RNA-binding domain of the nucleocapsid protein

2. **Supporting Tables**
- **Table S1.** Homology Model Statistics and Modeling Data
- **Table S2.** Site Nomenclature and Information 1 Homology Model Template PDB ID
- **Table S3**: 'Medium Confidence Hits' and Putative Mechanism of Hits with a z-score > 1.8 at < 10 sites
- **Table S4**: List of 132 Hits
- **Table S5**: Reference drugs identified using FQ score
- **Table S6**. Numbers of ions used in MD Simulations

3. **Supporting References**

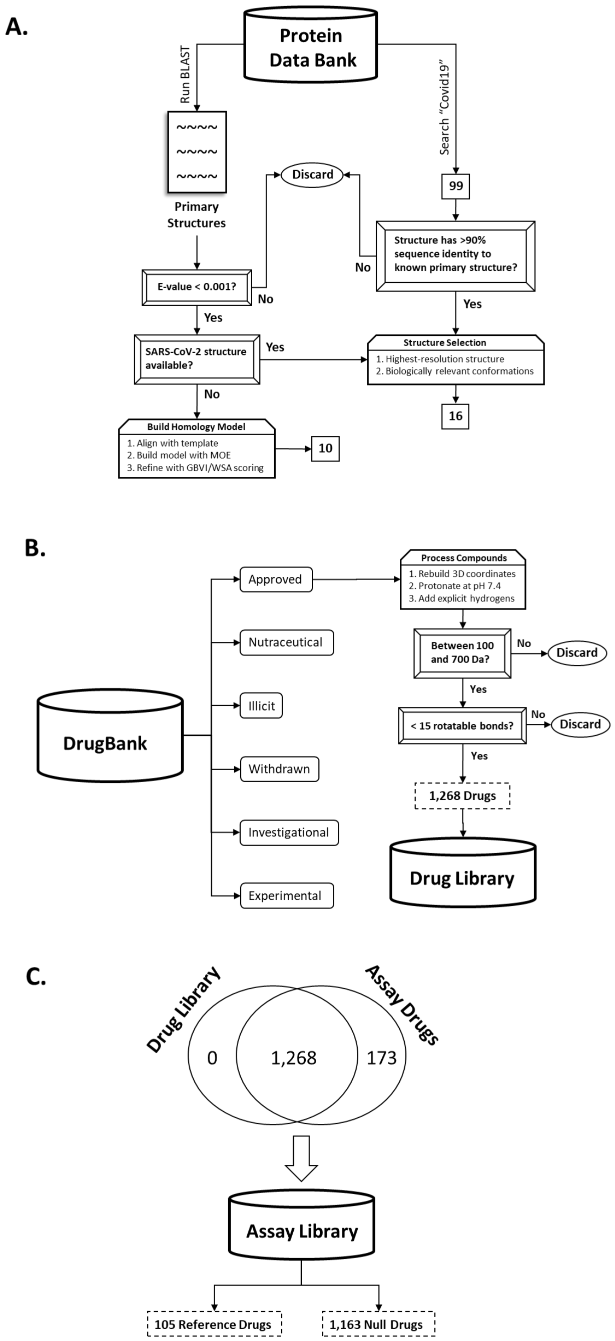

Fig. S1. Flowchart for preparation of SARS-CoV-2 protein structures and drug library for docking. (A) SARS-CoV-2 protein structures were selected from available crystal structures, and where unavailable, homology models were created from related coronaviruses. **(B)** FDA-approved drugs were extracted from DrugBank and prepared for downstream docking experiments. **(C)** From the assay library, 1,268 FDA-approved drugs overlapped with our *in silico* drug library, out of which 105 small molecule drugs had IC_{50} ≤ 2 μM against Huh7 cells. These were termed 'Reference Drugs.The flowcharts were prepared using PowerPoint software.

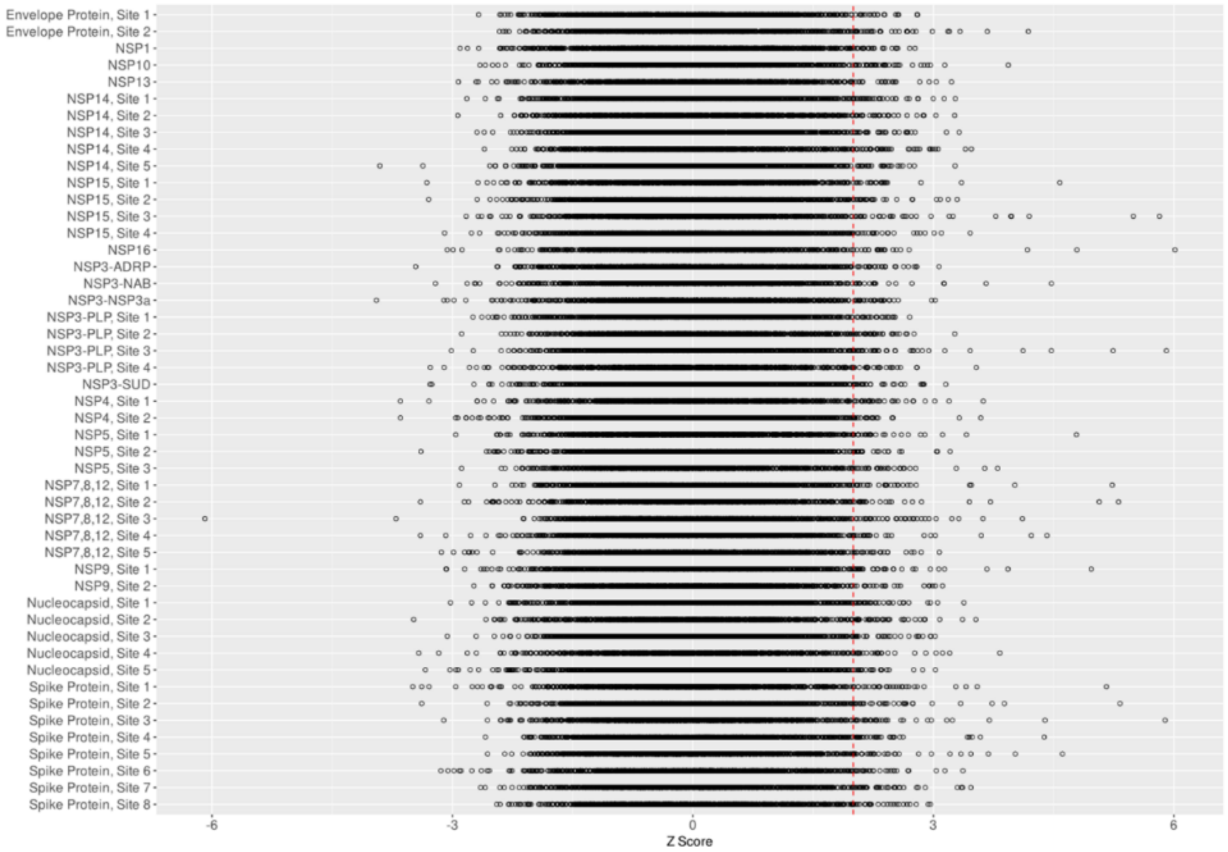

Fig. S2. Z score distributions of drugs for each predicted site. Each black open circle represents a single FDA-approved drug. The dashed red line depicts the Z score cutoff at 2. The plot was generated with R language using the ggplot2 package.

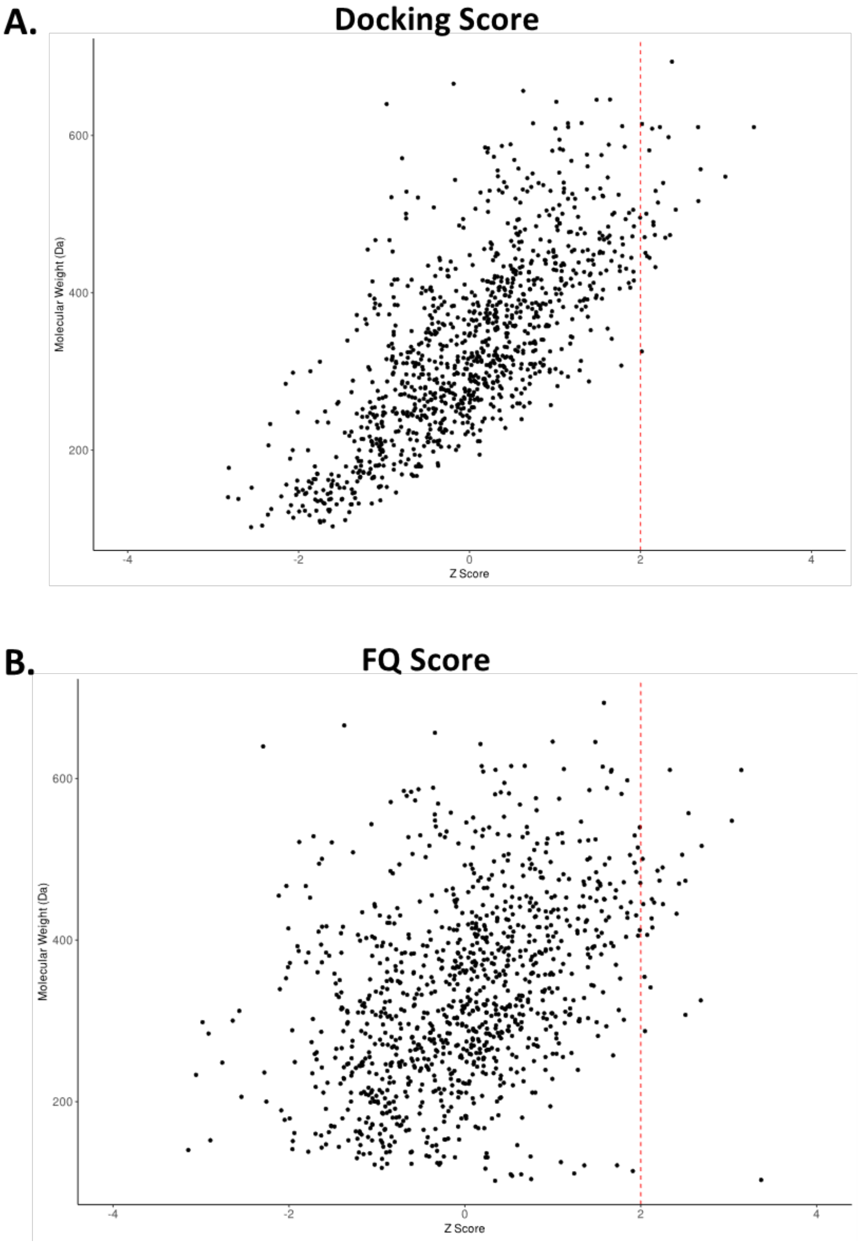

Fig. S3. Molecular weight dependence of scoring methods. The prioritized drugs from site 6 of the spike protein are shown using different scoring methods. (A.) A strong positive correlation was observed between the Z score and molecular weight for the prioritized drugs when using the docking score alone. (B.) However, utilizing the FQ score minimized the positive correlation. The dashed red line represents the Z score cutoff at 2. Plots were generated with R programming language and the ggplot2 package.

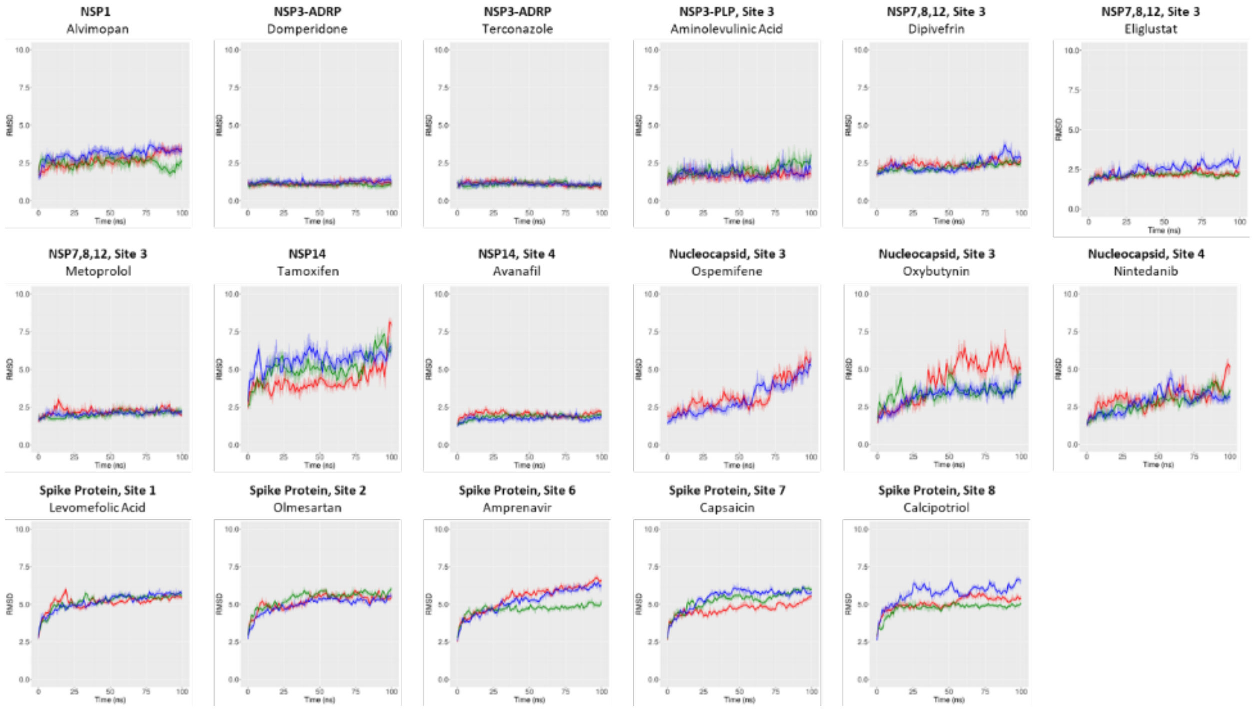

Fig. S4. Protein RMSDs from molecular dynamics simulations trajectories. RMSD calculations were performed using the peptide backbone (C, Cα, N). Runs 1, 2, and 3 are colored red, green, and blue, respectively. Systems with larger RMSD display the cases where drugs migrated to symmetry-related sites. Plots were generated with R programming language and the ggplot2 package.

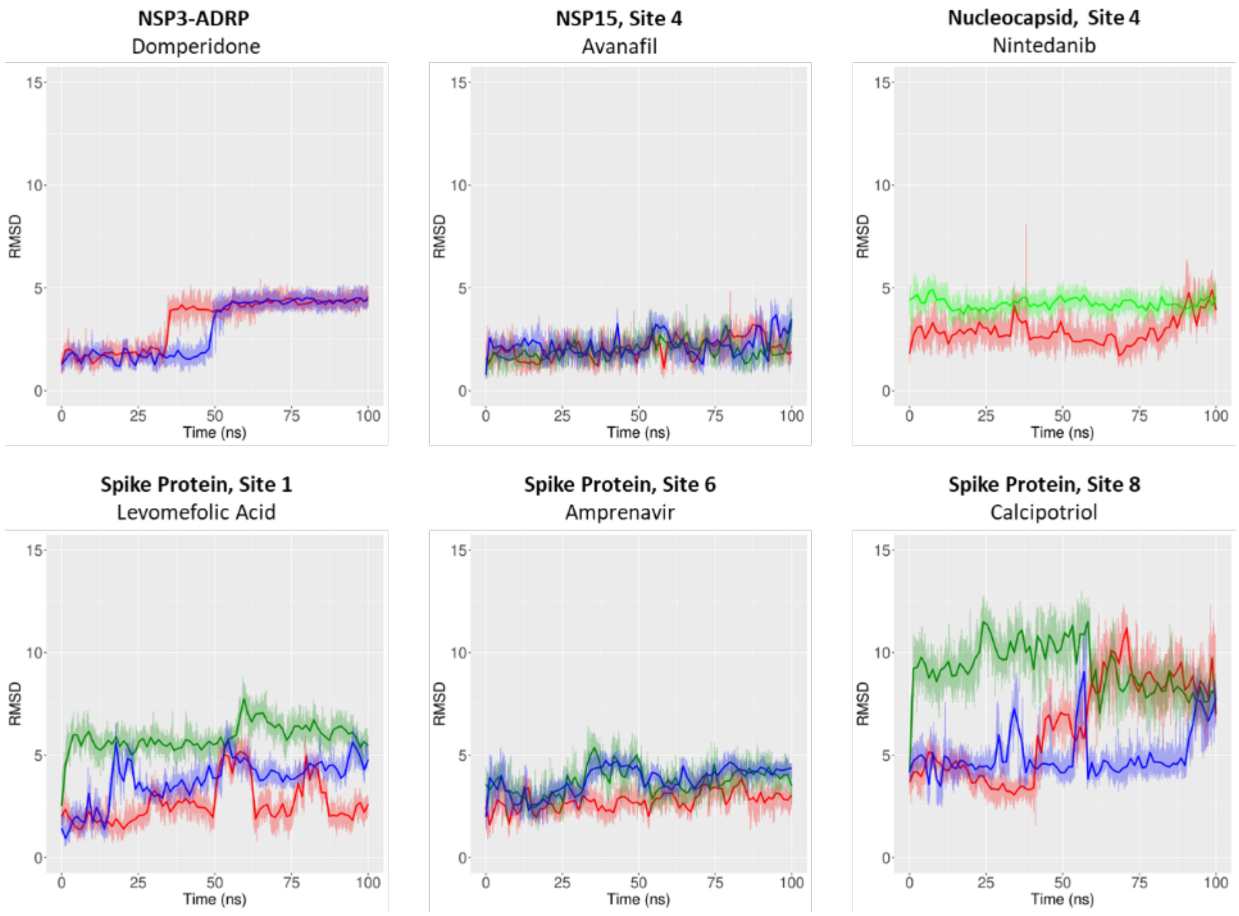

Fig. S5. **Drug RMSDs from trajectories of successful systems.** RMSD calculations were performed using the heavy atoms (i.e., non-hydrogen atoms) of the drug. Runs 1, 2, and 3 are colored red, green, and blue, respectively. Plots were generated with R programming language and the ggplot2 package.

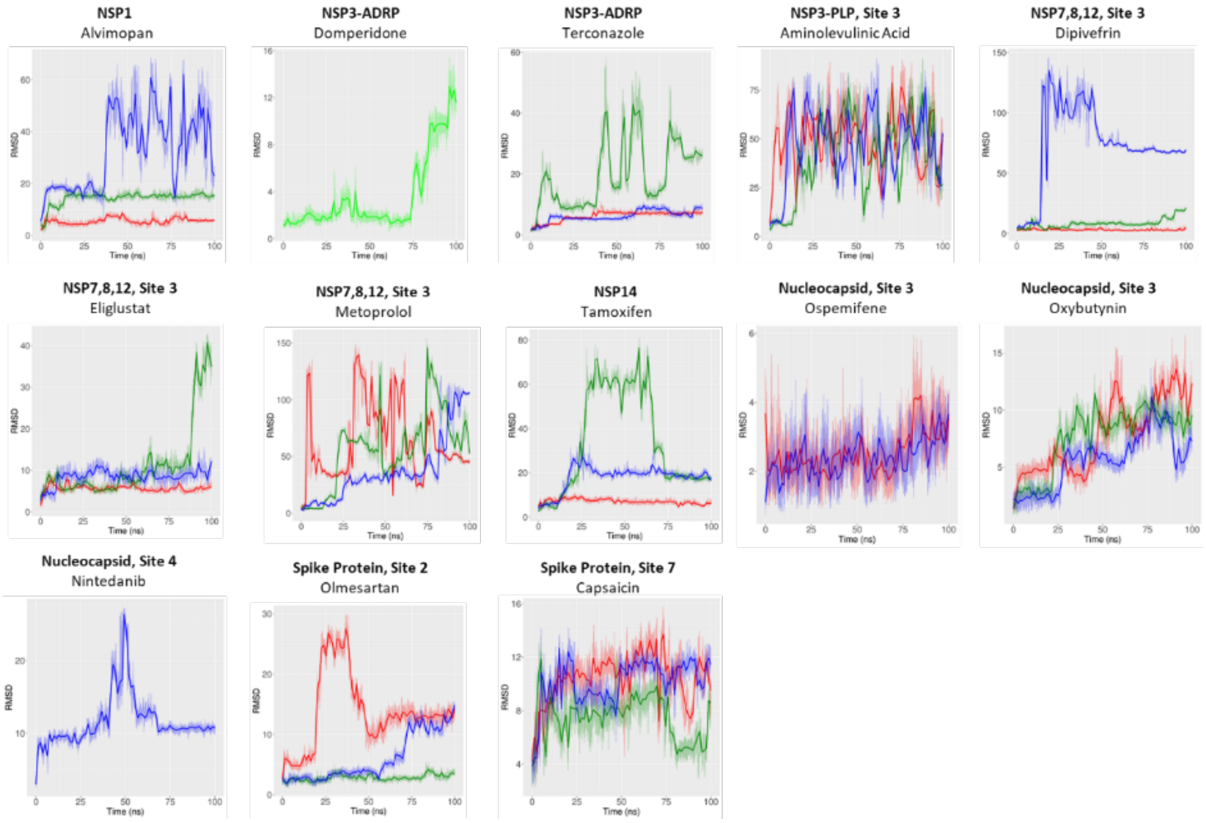

Fig. S6. Drug RMSDs from trajectories of failed systems. RMSD calculations were performed using the heavy atoms (i.e., non-hydrogen atoms) of the drug. Runs 1, 2, and 3 are colored red, green, and blue, respectively. Plots were generated with R programming language and the ggplot2 package.

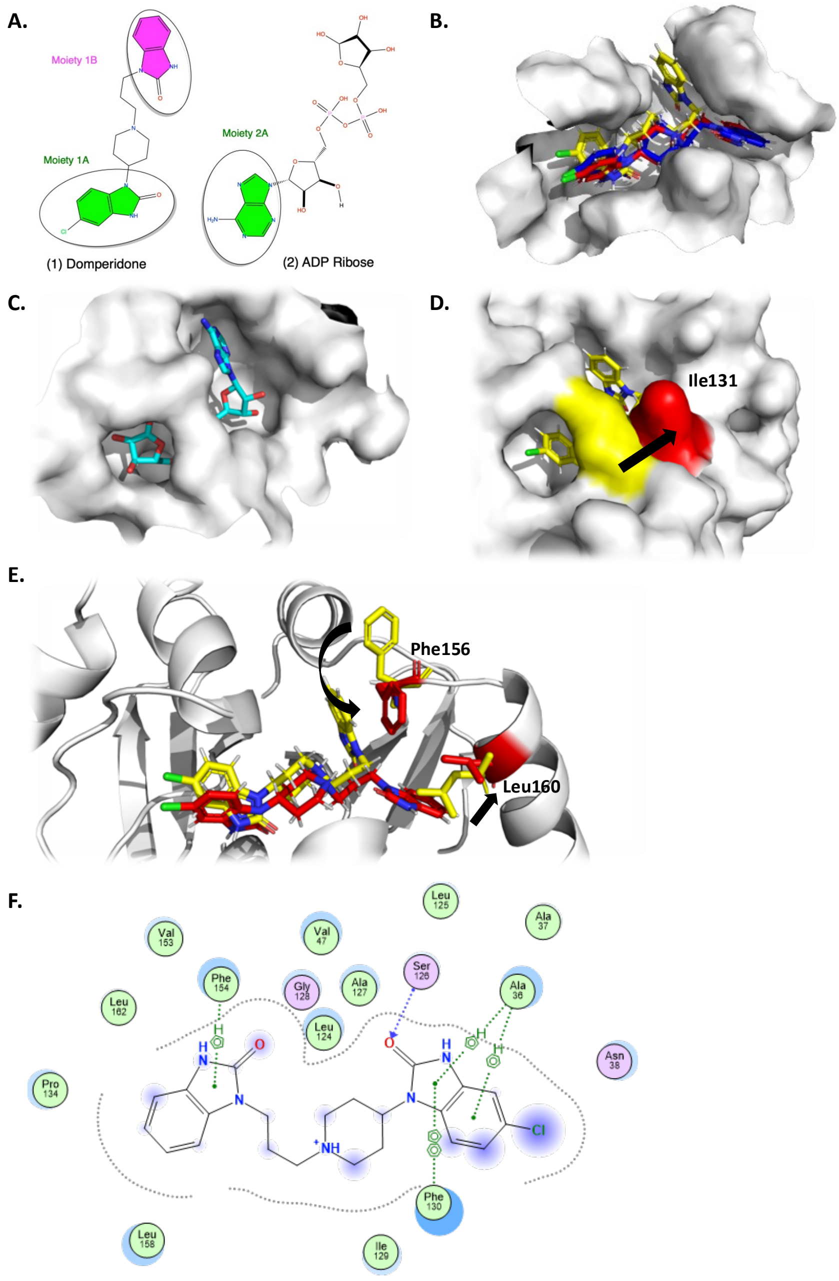

Fig. S7. Domperidone is predicted to displace ADP ribose from the ADRP domain of NSP3. (**A**) The chemical structures of domperidone (1) and ADP ribose (2), with Moiety 1. (**B**) The final snapshots from runs 1 and 3 (red and blue, respectively) are displayed in comparison with the original docked pose (yellow). (**C**) ADP ribose (cyan) binds to the active site of the ADRP domain of NSP3 (PDB: 6WOJ). (**D**) Upon achieving a stable interaction, domperidone was observed to cause Ile131 to move outwards, opening up the 'clamp' seen over the ribose on ADP ribose prior to hydrolysis. (**E**) Furthermore, Leu160 moves away to reveal a cryptic site that allows for the

accommodation of the non-chlorinated 1,3-benzodiazol-2-one group of domperidone, which is further stabilized with Phe156. For both (**D**) and (**E**), the original docked system (yellow) was compared with the final frame of run 1 (red). (**F**) A 2D interaction map of domperidone bound in the system with final frame of first MD simulation replicate. Graphics were generated using PyMOL and MOE.

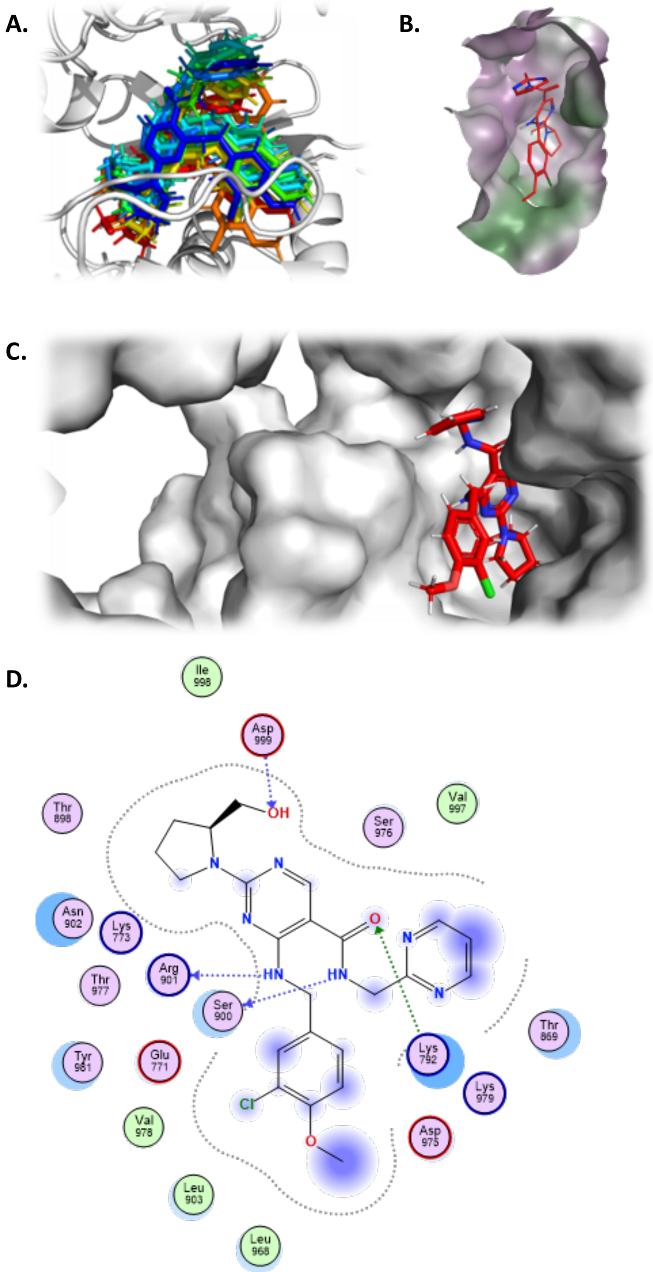

Fig. S8. Avanafil interacts with an allosteric site on NSP15. (**A**.) Representative frames from run 1 are shown in 10 ns increments (blue → green → red). (**B**.) Hydrophobic surface representation of the putative allosteric site to which avanafil binds was determined using MOE, where green and purple denote hydrophobicity and hydrophilicity, respectively. (**C.**) Avanafil is situated in an exposed pocket that is readily accessible to the exterior of the protein via a solvent channel. (**D.**) A 2D interaction map of avanafil with the system with final frame of first MD simulation replicate. Molecular graphics were generated using PyMOL and MOE.

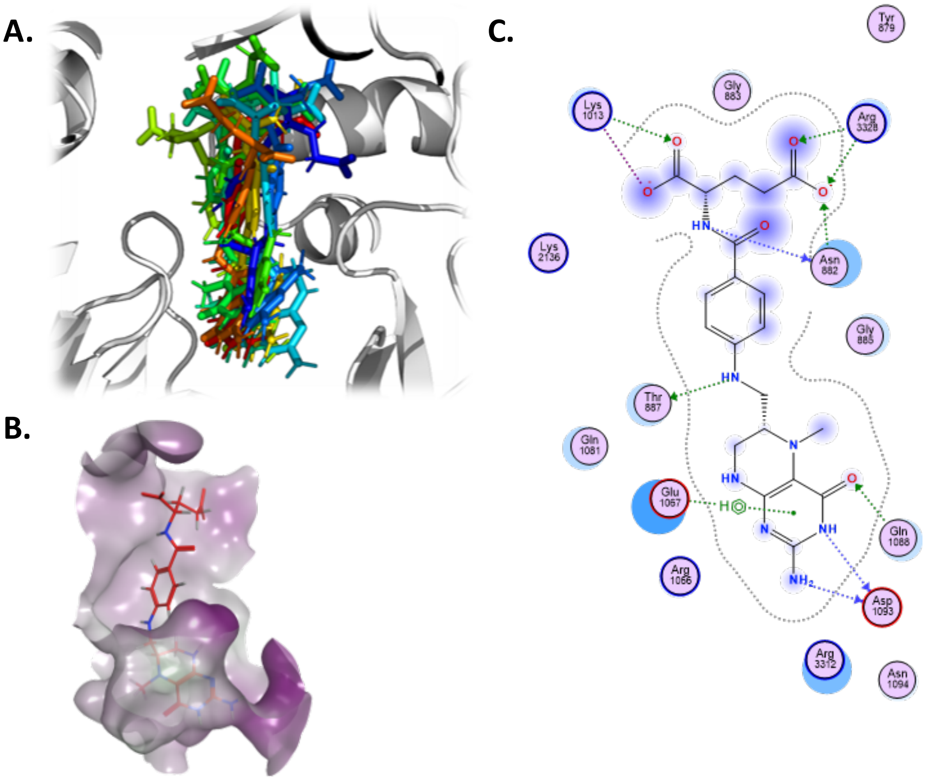

Fig. S9. **Levomefolic acid interacts with a putative allosteric site formed between the trimeric spike protein complex.** (**A.**) Representative frames from run 1 are shown in 10 ns increments (blue → green → red). (**B.**) Hydrophobic surface representation of the putative allosteric site to which levomefolic acid binds was determined using MOE, where green and purple denote hydrophobicity and hydrophilicity, respectively. (**C.**) A 2D interaction map of levomefolic acid with the system with final frame of first MD simulation replicate. Molecular graphics were generated using PyMOL and MOE.

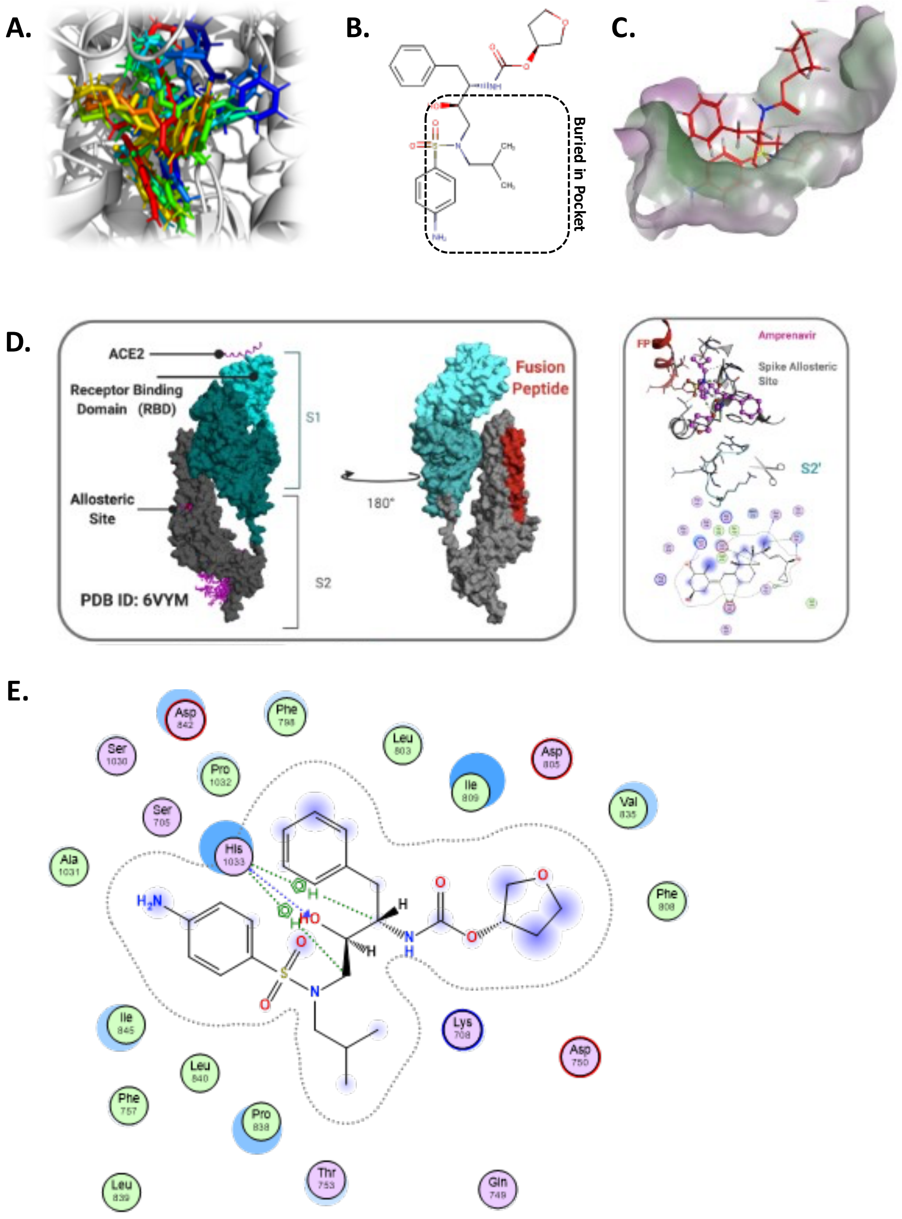

Fig. S10. Amprenavir interacts stably with a putative allosteric site on the spike protein. (A.) Representative frames from run 1 are shown in 10 ns increments (blue → green → red). (B.) Part of amprenavir remains buried in the predicted site throughout the simulations. MarvinSketch was used for drawing and displaying the 2D chemical structure, MarvinSketch 18.18.0, ChemAxon (https://www.chemaxon.com). (C.) Hydrophobic surface representation of the putative allosteric site to which amprenavir binds was determined using MOE, where green and purple denote hydrophobicity and hydrophilicity, respectively. (D.) (Left) Single monomer of homotrimer S protein. Domains colored RBD (Cyan), S1 (cyan marine). (Right) Amprenavir to spike allosteric site. 2D generation map of docked pose. Created with biorender.com. (E.) A 2D interaction map of amprenavir with the system with final frame of first MD simulation replicate. Molecular graphics were generated using PyMOL and MOE.

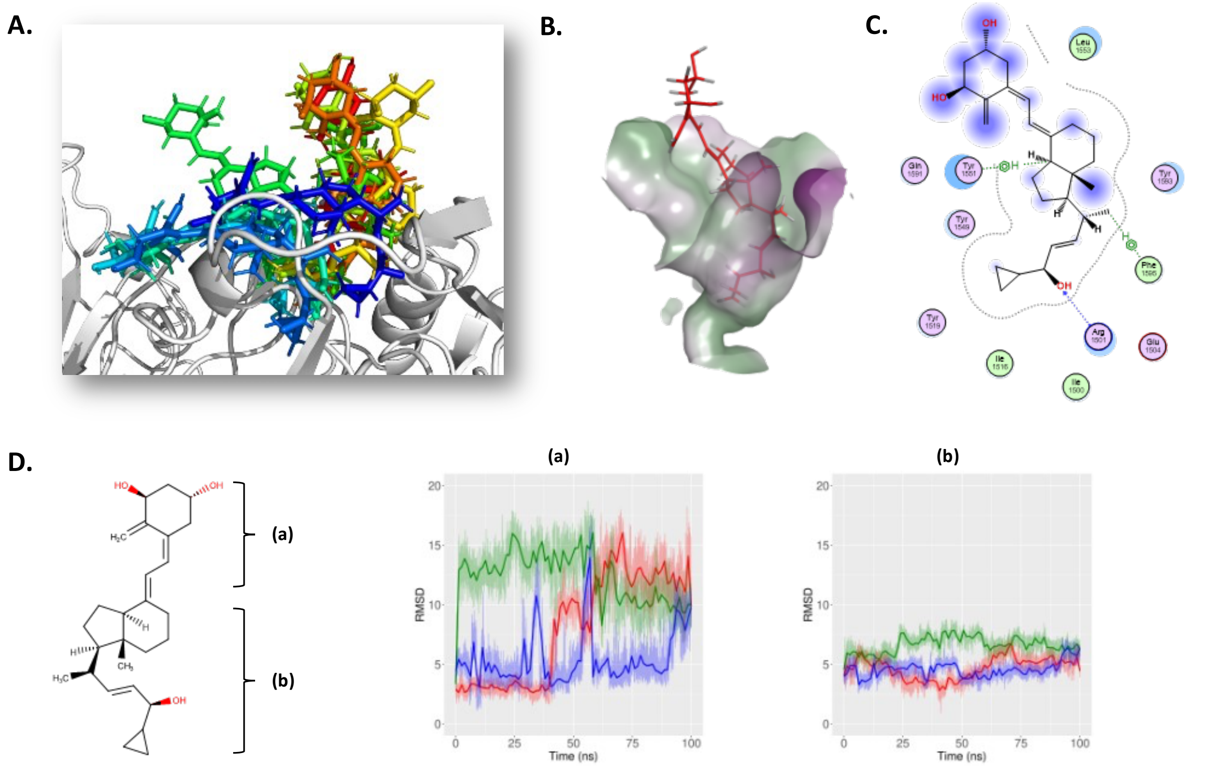

Fig. S11. Calcipotriol maintains a stable interaction with the RBD of the spike protein. (**A**.) Representative frames from run 1 are shown in 10 ns increments (blue → green → red). (**B**.) Hydrophobic surface representation of the putative allosteric site to which amprenavir binds was determined using MOE, where green and purple denote hydrophobicity and hydrophilicity, respectively. *(***C**.) A 2D interaction map of calcipotriol with the system. (D.) Calcipotriol contained both (a) solvent-exposed and (b) buried regions. The heavy atoms (i.e., non-hydrogen atoms) were used to calculate drug RMSDs from the trajectories for both regions individually. Runs 1, 2, and 3 are colored red, green, and blue, respectively. MarvinSketch was used for drawing and displaying the 2D chemical structure, MarvinSketch 18.18.0, ChemAxon (https://www.chemaxon.com). Molecular graphics were generated using PyMOL and MOE.

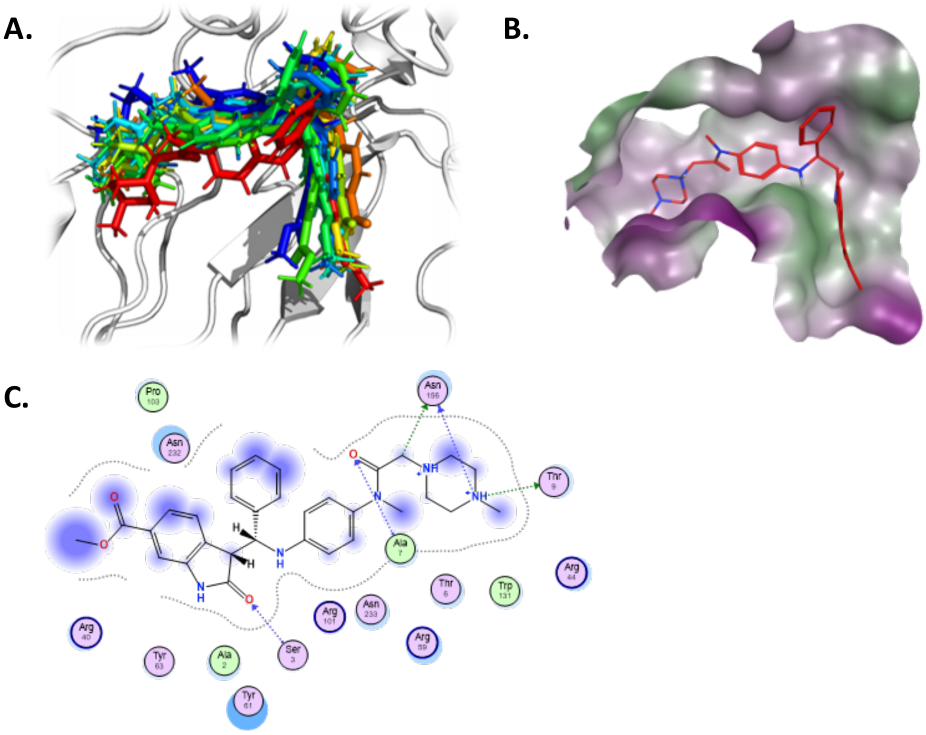

Fig.S12. **Nintedanib interacts with the RNA-binding domain of the nucleocapsid protein. (A)** Representative frames from run 1 are shown in 10 ns increments (blue → green → red). **(B)** Hydrophobic surface representation of the putative allosteric site to which nintedanib binds was determined using MOE, where green and purple denote hydrophobicity and hydrophilicity, respectively. **(C.)** A 2D interaction map of nintedanib with the system with final frame of first MD simulation replicate. Molecular graphics were generated using PyMOL and MOE.

Table S1. Homology Model Statistics and Modeling Data

Protein or Domain	Species	E-value	Template PDB ID	% ID	Region	RMSD to Mean	Cα RMSD to Mean	Contact Energy	Packing Score	GB/VI	U	E sol	E ele	E vdw	E bond	Atom Clashes	BB Bond Outliers	BB Angle Outliers	BB Torsion Outliers	Rotamer Outliers
**NSP1**	SARS	4.603e-60,	2GDT	86%	2-116	0.1601	0.00000	-106.62647	2.1425035	-32994	-8644	-1796	-9102	14	444	0	0	4	4	0
**NSP3a**	SARS	2.74E-48	2GRI	79%	17-112	0.0000	0.00000	-80.180763	2.2122338	-26454	-6145	-2470	-6852	217	491	9	0	1	2	0
**NSP3 SUD**	SARS	1.91E-126	2W2G	75%	1-264	0.0776	0.03018	-465.59048	2.2028725	-143896	-38098	-6419	-40012	-204	2117	8	3	20	13	0
**NSP3 NAB**	SARS	2.98E-49	2K87	82%	2-92	0.1494	0.00000	-61.182095	2.1671281	-22871	-5925	-1592	-6277	-19	371	0	0	2	4	0
**NSP 4**	Mouse Hepatitis A59	2.47E-23	3VCB	60%	5-94	0.4073	0.24649	-62.182476	2.1781962	-22020	-5164	-1031	-6574	292	1119	23	7	10	7	0
**NSP13 (dimer)**	SARS	0	6JYT	100%	3-603	0.0183131	0.007030345	-873.005	2.2819099	-468183	-112055	-18909	-119831	2262	5514	68	12	30	65	5
**NSP14**	SARS	0	5C8S	95%	1-527	0.286933	0.2587229	-809.2572	2.1527419	-383837	-91606	-19052	-99075	2099	5370	97	0	17	10	5
**E Protein (Pentamer)**	SARS	1.01E-23	5X29	89%	20-81	0.8426	0.76003	-118.68491	2.1943591	-1364	-245	-562	-513	22	245	115	4	7	19	3
**Nucleocapsid CTD**	SARS	4.65E-66	2CJR	96%	11-128	0.0000	0.00000	-258.84213	2.177073	-163763	-40916	-6551	-43357	435	2006	44	19	9	14	1

Table S2. Site nomenclature, sites, and PDB ID. Residues numbers to define sites.

Protein	Site #	"High-Confidence" Hits	Site Type	PDB ID	Co-Crystallized Ligand [PLB-Score]	Site Notes
NSP1	1	-	Putative Allosteric	2GDT*[1]	[1.9]	Site finder. N-terminal domain of protein. Residues: V56, E57, K58, G59, V60, L61, P62, G98, R99
NSP3a	*1*	-	Putative Allosteric	2GRI*[2]	[2.7]	Site finder. Residues: E11, L12, D13, E14, I62, E66, W67, A70, T71, Y72, Y73, C89, S90, F91 (2GRI Numbering)
NSP3 - ADRP	*1*	Domperidone, Terconazole	Co-Crystallized Ligand	6W02[3]	AMP (PDB ID: 6W02)[3]	AMP site. Residues: A21, D22, I23, V24, A38, A39, N40, K44, H45, G46, G47, G48, V49, A50, G51, A52, G97, P125, L126, L127, S128, A129, G130, I131, F132, G133, P136, A154, V155, F156, D157, L160 (6W02 Numbering)
NSP3 SUD	*1*	*Fedratinib*	*Nucleotide binding site*	2W2G*[4]	[2.8]	Site finder. Chain .B.Residues: G513, Q514, G515, L516, N517, G518, Y519, T520, V521, E523, S540, I541, I542, I617, N618, N621, R646, Y647, R649, S650, K652, S675, S676 (NSP3 numbering)
NSP3 PLPro	*1*	-	*Active Site/ Co-crystallized*	6W9C[5]	C0-ccrystalized Ligands (PDB ID:3MJ5, 3E9S*, 4OW0, 4OVZ)[9-10] [PLB1.9]	Protease active site. Residues: L162.A, G163.A, D164.A, V165.A, R166.A, E167.A, S170.A, Y171.A, M206.A, M208.A, S245.A, A246.A, P247.A, P248.A, Y264.A, N267.A, Y268.A, Q269.A, G271.A, Y273.A, T301.A, K105.B, W106.B, A107.B, D108.B
	2	*Aminovulinic Acid*	Putative Allosteric		[1.8]	Site finder. Residues: D12.A, N13.A, I14.A, Y56.A, Y71.A, Y72.A, Y83.A, A131.A, D134.A, A135.A, R138.A, E143.A, N146.A, L150.A
	3	-	*Co-Crystalized Ligand*		Co-crystallized ligands (PDB ID: 6Y7M)[6]	Residues: A39.A, D40.A, V41.A, T42.A, K43.A, I44.A, S85.A, N88.A, H89.A, K92.A, W93.A, K94.A, K105.A, W106.A, A107.A, D108.A, N109.A, V159.A, G160.A, E161.A, L162.A, Q269.A, D108.B, N109.B, C155.B, N156.B, K157.B, T158.B, V159.B, G160.B, E161.B, L162.B, G163.B, D164.B, R166.B, E167.B, Y171.B, M208.B, S245.B, A246.B, P247.B, P248.B, Y264.B, N267.B, Y268.B, Q269.B, C270.B, G271.B, Y273.B, T301.B, D302.B, D76.C, P77.C, S78.C, R82.C, H89.C, K92.C, W93.C, K94.C, I104.C, K105.C, W106.C, A107.C, D108.C, N109.C, N110.C, Y112.C, C155.C, N156.C, K157.C, T158.C, V159.C, G160.C, E161.C, L162.C, G163.C, D164.C, R166.C, E167.C, T168.C, Y171.C, M208.C, A246.C, P247.C, P248.C, Y264.C, N267.C, Y268.C, Q269.C, Y273.C, L289.C, T301.C, D302.C
NSP3 NAB	*1*	-	*RNA Binding Site?*	2K87*[7]	[1.6]	Site finder. Residues: K93, K117, P118, T120, W121, R124
NSP4	1	-	Putative Allosteric	3VCB*[8]	[2.8]	Site finder. Residues: Y448, Y451, K452, F454, M458, Y463, A466, C469, H470, P490, Q491
	2	-	Putative Allosteric		[1.1]	Site finder. Residues: Y448, Y451, K452, Y453, F454, S455, M458, Y463, A466, A467, H470, K473, A474, D477, L486, Y487, Q488, P489, P490, Q491, T492
NSP5	1	Fosamprenavir	Putative Allosteric	6YB7[11]	[4.5]	Site finder. Near crystal homomeric dimer face. Residues:F3.A, R4.A, K5.A, M6.A, A7.A, V125.A, Y126.A, Q127.A, R131.A, K137.A, G138.A, T169.A, G170.A, V171.A, H172.A, D197.A, T198.A, T199.A, W207.A, L282.A, G283.A, S284.A, A285.A, L286.A, L287.A, E288.A, D289.A, E290.A, F291.A, S1.B, G2.B, F3.B, R4.B, K5.B, M6.B, A7.B, V125.B, Y126.B, Q127.B, C128.B, R131.B, K137.B, D197.B, T198.B, T199.B, W207.B, N214.B, D216.B, L282.B, G283.B, S284.B, A285.B, L286.B, L287.B, E288.B, D289.B, E290.B, F291.B
	2	-	Classical (Active Site)		Co-crystallized ligands (e.g., 5RG0,5RFZ,5RFI)[12] [PLB = 3.7]	Cys protease active site. Co-crystallized ligands (e.g., 5RG0,5RFZ,5RFI) Residues: T24.A, T25.A, T26.A, L27.A, N28.A, H41.A, V42.A, C44.A, T45.A, S46.A, E47.A, M49.A, P52.A, Y54.A, Y118.A, F140.A, L141.A, N142.A, G143.A, S144.A, C145.A, H163.A, H164.A, M165.A, E166.A, H172.A, D187.A, R188.A, Q189.A
	3	Eliglustat, Fedratinib	Co-Crystalized Ligand		Co-crystallized ligands (PDB ID: 5RE6, 5RE5, 5RFC).	Residues: V35.A, E55.A, D56.A, L58.A, I59.A, N63.A, I78.A, G79.A, H80.A, S81.A, M82.A, K88.A, K90.A
NSP 7/8/12	1 (NSP12)	-	Classical (RNA Binding Site)	6M71[13]	[4.4]	Remdesivir docked in predicted pose. Residues: Q444.A, A448.A, S451.A, D452.A, Y455.A, M542.A, K545.A, K551.A, N552.A, R553.A, A554.A, R555.A, T556.A, V557.A, G616.A, W617.A, D618.A, Y619.A, P620.A, K621.A, C622.A, D623.A, R624.A, S682.A, D760.A, D761.A, A762.A, V763.A, A797.A, K798.A, C799.A, W800.A, H810.A, E811.A, F812.A, C813.A, S814.A, Q815.A
	2 (NSP12)	-	Putative Allosteric		[5.5]	Large groove near helicase domain. Perhaps for RNA binding? Residues: L245.A, T246.A, R249.A, A250.A, L251.A, T252.A, S255.A, Y265.A, I266.A, W268.A, N314.A, V315.A, L316.A, S318.A, T319.A, V320.A, F321.A, P322.A, P323.A, T324.A, F326.A, G327.A, P328.A, V330.A, S343.A, Y346.A, H347.A, F348.A, R349.A, E350.A, P378.A, A379.A, A382.A, A383.A, T394.A, C395.A, F396.A, V398.A, Y456.A, R457.A, N459.A, L460.A, P461.A, T462.A, P537.A, P627.A, N628.A, M629.A, S664.A, M666.A, V675.A, K676.A, P677.A, G678.A, P116.D, L117.D, N118.D, I119.D, I120.D, P121.D, L122.D
	3 (NSP7)	Metoprolol, Dipivefrin, Eliglustat	PPI (NSP 7-8)		-	Interface between NSP7 and NSP8. Residues: L56.B, L59.B, L60.B, M62.B, Q63.B, G64.B, A65.B, V66.B, D67.B, I68.B, F92.C, I107.C, V115.C
	4 (NSP8)	-	Putative Allosteric		[1.8]	Site finder. Residues: I106.C, N109.C, A110.C, C114.C, V115.C, P116.C, I120.C, L128.C, V130.C, V131.C, I132.C
	5 (NSP7)	-	PPI (NSP7-12)		-	Interface between NSP7 and NSP12. Residues: T409.A, V410.A, K411.A, P412.A, G413.A, N414.A, F415.A, Y420.A, F429.A, E431.A, L437.A, K438.A, F440.A, F441.A, F442.A, A443.A, Q444.A, D445.A, G446.A, A550.A, K551.A, N552.A, F843.A
NSP9	1	-	Putative Allosteric	6W4B[14]	[2.1]	Site finder. Perhaps ssRNA binding site? Residues: Q50.B, D51.B, L52.B, K53.B, L70.B, E71.B, P72.B, P73.B, Y90.B
	2	-	Homodimer PPI and RNA Binding Site		[1.9]	Site finder. Likely groove for RNA binding, site also overlaps with homodimer interface. Residues: E71.A, P72.A, P73.A, C74.A, L89.A, Y90.A, F91.A, R100.A, L104.A, M1.B, N3.B, L5.B, L98.B
NSP10/16	1 (NSP10)	Fedratinib	Putative Allosteric	6W4H[15]	[2.5]	Site finder. Interaction site with NSP16. Residues: I4308, C4326, C4327, L4328, Y4329, H4336, P4337, N4338, F4342, C4343, D4344, L4345, V4361, T4364, L4365, N4367, T4368, V4369, W4376 *Residues as asssinged in PDB file*
	2 (NSP16)	-	Classical (Active Site)		S-Adenosylmethionine (SAM) [5.0]	Residues: K6822, C6823, D6824, L6825, Y6828, M6840, N6841, K6844, F6868, G6869, A6870, G6871, S6872, D6873, K6874, S6896, D6897, L6898, N6899, G6911, D6912, C6913, A6914, V6916, D6928, M6929, Y6930, D6931, P6932, T6934, K6935, V6937, G6946, F6947, T6949, Y6950, K6968, T6970, E6971, H6972, S6973, N6996, S6998, S6999, S7000, E7001 *Residues as asssinged in PDB file*
NSP13	1	-	Co-crystallized Ligands	6JYT*[16]	SSYA-001-10 and Adenosine [3.7]	Residues: K139, E142, E143, F145, K146, Y149, L158, R161, E162, P175, L176, N177, R178, N179, Y180, V181, T183, Q194, E197, Y198, T199, E201, K202, G203, D204, Y205, A208, V210, R212, T214, T228, H230, T231, M233, A308, C309, S310, H311, A312, A313, P335, R337, A338, R339, V340, T359, V360, N361, M378, A379, T380, Y382, D383, V386, R390, L405, P406, A407, P408, R409, T410, L411, L412, T413, G415, T416, L417, F422, S486, P514, Y515, N516, N519, A520, T530, Q531, T532, D534, S535, Q537, S539, H554, N557, N559, R560
NSP14	1	-	Co-crystallized Ligands	5C8S*[17]	Co-crystalized Ligands S-Adenosyl-L-Homocysteine and Guanosine-P3-	Residues: R289, V290, W292, R310, Q313, D331, I332, G333, N334, P335, Y351, D352, A353, Q354, P355, E365, L366, F367, Y368, S369, F384, W385, N386, C387, V389, F426
	2	-	Puttative Allosteric		Co-crystalized Ligands Guanosine-P3-Adenosine-5',5'-Triphosphate	Residues: I305, N306, A307, A308, C309, R310, K311, V312, Q313, H314, N334, K336, C340, W385, N386, C387, N388, F401, Y420, N422, K423, H424, F426, H427, T428, F506
	3	-	PPI (NSP10)		[3.9]	Site finder. Residues: Y124, T131, D132, F133, N160, R163, K196, V199, K200, F217, S218, T219, A220, S221, D222, D234, Y235
	4	-	Putative Allosteric		[1.6]	Site finder. Residues: P467, L468, K469, S470, A471, C473, I474, T475, N478, G481, A482, V483, C484, H487, A488, Y491, W520
	5	Tamoxifen	Classical (Active Site)		[3.7]	Putative catalytic site with residues numbered for SARS-CoV-2 homology model. Residues: I80, C285, F286, K288, R289, V290, D291, Y296, H424, A425
NSP15	1	Fosamprenavir	Classical (Active Site)	6W01[18]	Docked Benzopurin B	Ligand binding site. Residues:G230.A, Y231.A, A232.A, E234.A, H235.A, I236.A, V237.A, Y238.A, G239.A, D240.A, F241.A, S242.A, H243.A, S244.A, Q245.A, L246.A, G247.A, G248.A, L249.A, H250.A, L251.A, S289.A, K290.A, C291.A, V292.A, C293.A, S294.A, W333.A, K335.A, H338.A, V339.A, E340.A, T341.A, F342.A, Y343.A, P344.A, K345.A, L346.A, Q347.A
	2	-	Putative Allosteric		[4.2]	Site finder. Residues: E69.A, K71.A, K90.A, Q160.A, G165.A, V166.A, T167.A, L168.A, I169.A, T196.A, Q197.A, S198.A, R199.A, N200.A, L201.A, Q202.A, E203.A, F204.A, K205.A, R207.A, L252.A, L255.A, F259.A, F264.A, L266.A, D268.A, M272.A, D273.A, S274.A, T275.A, V276.A, K277.A, Y279.A, V295.A, I296.A, D297.A
	3	Raloxifene	PPI		[5.2]	Interface between two protomers .A and .B. Residues: S26.B, I28.B, N29.B, K35.B, G38.B, V39.B, D40.B, N30.C, N46.C, K47.C, T48.C, T49.C, L50.C, P51.C, V52.C, V54.C, L58.C, D79.C, I80.C, V85.C, K90.C, R91.C, D92.C, A93.C, P94.C, H96.C, I97.C, S98.C, T99.C, S104.C, M105.C, T106.C, D107.C, I108.C, T115.C, I116.C, P119.C, L120.C, P271.C, N30.D, N46.D, T48.D, T49.D, L50.D, P51.D, V52.D, V54.D, D79.D, I80.D, R91.D, I97.D, S98.D, T99.D, M105.D, T106.D, D107.D, I108.D, I116.D, C117.D, P119.D, L120.D, S26.E, I28.E, N29.E, Y33.E, K35.E, G38.E, V39.E, D40.E, V41.E, E42.E
	4	Avanafil	Putative Allosteric		[2.8]	Site Finder. Near interface between two PPIs, but not directly on it, may be allosteric. Residues :E261.A, S262.A, P263.A, E265.A, A284.A, Q285.A, E69.C, K71.C, K90.C, Q160.C, G165.C, V166.C, T167.C, L168.C, I169.C, E171.C, T196.C, Q197.C, S198.C, R199.C, N200.C, L201.C, Q202.C, E203.C, F204.C, K205.C, R207.C, L252.C, L255.C, F259.C, F264.C, E265.C, L266.C, D268.C, M272.C, D273.C, S274.C, T275.C, V276.C, K277.C, Y279.C, V295.C, I296.C, D297.C
S - Spike	1	-	Putative Allosteric or PPI	6VYB[19]	[10.5]	Site Finder. Near homomer interface. Residues: I712.A, A713.A, I714.A, P715.A, S884.A, G885.A, W886.A, T887.A, L894.A, Q895.A, I896.A, Q901.A, Y904.A, N907.A, G908.A, I909.A, G910.A, V911.A, T912.A, Q913.A, N914.A, E1031.A, G1035.A, Q1036.A, S1037.A, K1038.A, R1039.A, V1040.A, D1041.A, G1046.A, Y1047.A, H1048.A, K1086.A, H1088.A, F1089.A, P1090.A, R1091.A, E1092.A, G1093.A, V1094.A, V1104.A, T1105.A, Q1106.A, R1107.A, N1108.A, E1111.A, Q1113.A, I1114.A, T1116.A, T1117.A, D1118.A, N1119.A, T1120.A, F1121.A, V1122.A, S1123.A, G1124.A, V1137.A, D1139.A, P1140.A, L1141.A, Q1142.A, E1144.A, L1145.A, I712.B, A713.B, I714.B, P715.B, S884.B, G885.B, W886.B, T887.B, A890.B, L894.B, Q895.B, I896.B, Q901.B, Y904.B, R905.B, F906.B, N907.B, G908.B, I909.B, G910.B, V911.B, T912.B, Q913.B, N914.B, E918.B, G1035.B, Q1036.B, S1037.B, K1038.B, R1039.B, V1040.B, D1041.B, F1042.B, C1043.B, G1044.B, K1045.B, G1046.B, Y1047.B, H1048.B, T1066.B, H1083.B, K1086.B, H1088.B, P1090.B, R1091.B, E1092.B, G1093.B, V1094.B, V1104.B, T1105.B, Q1106.B, R1107.B, N1108.B, F1109.B, E1111.B, Q1113.B, I1114.B, T1116.B, T1117.B, D1118.B, N1119.B, T1120.B, F1121.B, V1122.B, S1123.B, D1139.B, P1140.B, L1141.B, E1144.B, I712.C, A713.C, I714.C, P715.C, S884.C, G885.C, W886.C, T887.C, L894.C, Q895.C, I896.C, Q901.C, Y904.C, R905.C, N907.C, G908.C, I909.C, G910.C, V911.C, T912.C, Q913.C, N914.C, E1031.C, G1035.C, Q1036.C, S1037.C, K1038.C, R1039.C, V1040.C, D1041.C, G1046.C, Y1047.C, H1048.C, K1086.C, H1088.C, P1090.C, R1091.C, E1092.C, G1093.C, V1094.C, V1104.C, T1105.C, Q1106.C, R1107.C, N1108.C, E1111.C, Q1113.C, I1114.C, T1116.C, T1117.C, D1118.C, N1119.C, T1120.C, F1121.C, V1122.C, S1123.C, V1137.C, Y1138.C, D1139.C, P1140.C, L1141.C, Q1142.C, E1144.C
	2	-	Putative Allosteric or PPI		[4.9]	Site Finder. PPI between heads of two trimers, though in open state. Near homomer interface. Residues: R319.A, S325.A, I326.A, V327.A, W353.A, N354.A, R355.A, K356.A, R357.A, Y380.A, G381.A, K386.A, D389.A, L390.A, C391.A, F392.A, Y396.A, P412.A, P426.A, D427.A, D428.A, F429.A, T430.A, K462.A, P463.A, F464.A, E465.A, R466.A, D467.A, I468.A, S514.A, F515.A, E516.A, L517.A, L518.A, H519.A, K528.A, N540.A, F541.A, N542.A, N544.A, G545.A, L546.A, T547.A, G548.A, T549.A, F565.A, R567.A, D568.A, I569.A, A570.A, D571.A, T572.A, T573.A, I587.A, P589.A, D40.B, V42.B, R44.B, F86.B, N87.B, D88.B, G89.B, G107.B, T108.B, T114.B, Q115.B, V130.B, T167.B, F168.B, N196.B, I197.B, D198.B, G199.B, Y200.B, P230.B, I231.B, G232.B, I233.B, N234.B, I235.B, T734.B, M740.B, Y741.B, I742.B, C743.B, G744.B, D745.B, S746.B, K854.B, F855.B, N856.B, L858.B, T859.B, V860.B, L959.B, N960.B, V963.B, L966.B, S967.B, N969.B, G971.B, A972.B, I973.B, S974.B, S975.B, V976.B, L977.B, N978.B, D979.B, L981.B, S982.B, R983.B, Q992.B, R995.B, L996.B, R1000.B, L752.C, Q755.C, E990.C, V991.C, D994.C
	3	-	Putative Allosteric		[3.6]	Site Finder. Residues: D40.A, K41.A, V42.A, F43.A, R44.A, S45.A, S46.A, V47.A, L48.A, H49.A, S50.A, Q52.A, K202.A, R273.A, T274.A, L276.A, C291.A, A292.A, E298.A, C301.A, T302.A, L303.A, K304.A, Y313.A, Q314.A, T315.A, S316.A, N317.A, D950.A, N953.A, Q954.A, Q957.A, A958.A, N960.A, T961.A, K964.A, Q965.A, S967.A, S968.A, N969.A, F970.A, I973.A, S974.A, S975.A, V976.A, D979.A, S982.A, R983.A, Q1010.A, I1013.A, R1014.A, A1016.A, E1017.A, V736.B, D737.B, C738.B, T739.B, M740.B, L754.B, Q755.B, Y756.B, G757.B, S758.B, C760.B, T761.B, Q762.B, N764.B, R765.B, A766.B, T768.B, G769.B, I770.B, V772.B, E773.B, K776.B, L1012.B, A1015.B, A1016.B, R1019.B, L390.C, C391.C, F392.C, L517.C, L518.C, H519.C, A520.C, N544.C, G545.C, L546.C, K557.C, F565.C, G566.C, R567.C, D568.C, I569.C, A570.C, D571.C, D574.C
	4	-	PPI? (Trimer)		[2.9]	Site Finder. Center core of trimer Spike protein. Near homomer interface. Residues: E725.A, L727.A, P728.A, E773.A, K776.A, N777.A, E780.A, K947.A, D950.A, V951.A, Q954.A, R1014.A, A1016.A, E1017.A, I1018.A, R1019.A, A1020.A, S1021.A, N1023.A, L1024.A, A1026.A, T1027.A, K1028.A, S1030.A, E1031.A, R1039.A, V1040.A, D1041.A, F1042.A, G1044.A, E725.B, L727.B, P728.B, E773.B, K776.B, N777.B, E780.B, G946.B, K947.B, D950.B, V951.B, A1016.B, E1017.B, I1018.B, R1019.B, A1020.B, S1021.B, N1023.B, L1024.B, A1026.B, T1027.B, K1028.B, S1030.B, E1031.B, R1039.B, V1040.B, D1041.B, F1042.B, E725.C, L727.C, P728.C, K776.C, E780.C, Q784.C, K947.C, D950.C, V951.C, Q954.C, R1014.C, A1016.C, E1017.C, I1018.C, R1019.C, A1020.C, S1021.C, N1023.C, L1024.C, A1026.C, T1027.C, K1028.C, S1030.C, E1031.C, R1039.C, V1040.C, D1041.C, F1042.C, G1044.C
	5	-	Putative Allosteric		[2.4]	Site Finder. Closed region of RBD domain. Similar to Site 7. Residues: T345.A, R346.A, F347.A, V401.A, L441.A, D442.A, V445.A, G446.A, N448.A, N450.A, Y451.A, R509.A
	6	-	Putative Allosteric		[2.3]	Site Finder. Near S2' cleavage site. Residues: V729.A, S730.A, M731.A, T732.A, K733.A, T778.A, F782.A, F823.A, P863.A, L865.A, T866.A, D867.A, I870.A, S1055.A, A1056.A, P1057.A, H1058.A, G1059.A
	7	-	PPI		[2.0]	Site Finder. Closed conformation near RBD and spike homomer interface. Residues: F342.A, N343.A, A344.A, T345.A, F347.A, S371.A, S373.A, F374.A, W436.A, N437.A, S438.A, N439.A, N440.A, L441.A, D442.A, R509.A
	8	-	Classical (PPI w/ ACE2)		ACE2 (PDB ID: 6VW1)[20]	Open Conformation, RBD contacts w/ ACE2. Residues: R403.B, E406.B, K417.B, N439.B, S443.B, K444.B, V445.B, G446.B, G447.B, N448.B, Y449.B, L452.B, Y453.B, R454.B, L455.B, F456.B, R457.B, K458.B, L492.B, Q493.B, S494.B, Y495.B, F497.B, Q498.B, P499.B, T500.B, N501.B, G502.B, V503.B, G504.B, Y505.B, Q506.B
E Protein	1	-	PPI	5X29*[21]	[2.2]	Site Finder. Core of pentamer, would inihibt oligimerization. Residues: F23.A, L27.A, T30.A, L31.A, I33.A, L34.A, L39.A, Y42.A, C43.A, N45.A, I46.A, V47.A, S50.A, L51.A, P54.A, Y57.A, V24.B, V25.B, L28.B, V29.B, L31.B, A32.B, I33.B, T35.B, A36.B, L37.B, R38.B, C44.B, V47.B
	2	-	PPI		[2.2]	Site Finder. Near PPI of homodimer. Residues: L18.A, L19.A, A22.A, V25.A, F26.A, N15.B, L18.B, L19.B, A22.B, F26.B, V29.B, T30.B, I33.B, L34.B, E8.C, T9.C, G10.C, L18.C, L19.C, A22.C, F23.C, F26.C, L27.C, V29.C, T30.C, L31.C, I33.C, L34.C, L39.C, Y42.C, C43.C, I46.C, V47.C, S50.C, L51.C, P54.C, E8.D, T9.D, L12.D, V14.D, N15.D, S16.D, L18.D, L19.D, A22.D, V25.D, F26.D, V29.D, A32.D, I33.D, T35.D, A36.D, L37.D, R38.D, E8.E, L18.E, L19.E, L21.E, A22.E, F23.E, F26.E
Nucleocapsid	1	-	PPI	6VYO[22]	[3.8]	Site finder. All sites at or near PPI interfaces, which could disrupt packaging of RNA into nucelocapsid. Residues:N48.A, T49.A, A50.A, W52.A, N75.A, T76.A, N77.A, G114.A, T115.A, G116.A, K143.A, D144.A, H145.A, I146.A, G147.A, T148.A, R149.A, N150.A, N153.A, N154.A, A155.A, A156.A, I157.A, T54.D, A55.D, L56.D, T57.D, H59.D, R92.D, I94.D, R95.D, G96.D, G97.D, K102.D, L104.D, S105.D, R107.D, Y109.D, V158.D, L159.D, A173.D
	2	-	PPI		[2.8]	Site finder. Residues: F53.B, T54.B, A55.B, L56.B, T57.B, H59.B, R92.B, I94.B, L104.B, S105.B, R107.B, Y109.B, R149.B, A156.B, V158.B, L159.B, Y172.B, A173.B, G46.C, N47.C, N48.C, T49.C, W52.C, N77.C, T115.C, G116.C, H145.C, I146.C, G147.C, T148.C, R149.C, N150.C, N154.C
	3	-	RNA Binding Pore/PPI		[4.6]	Site finder. Overlaps between dimer interface and RNA binding site (PDB ID: 7ACS and 7ACT). Residues: A50.A, S51.A, F53.A, T54.A, A55.A, L56.A, T57.A, H59.A, R88.A, R92.A, I94.A, L104.A, S105.A, R107.A, Y109.A, Y111.A, P117.A, R149.A, A156.A, I157.A, V158.A, L159.A, Q160.A, L161.A, P162.A, Q163.A, G164.A, T165.A, L167.A, A173.A, G46.B, N47.B, N48.B, T49.B, W52.B, F53.B, P73.B, I74.B, N75.B, T76.B, N77.B, S78.B, S79.B, D82.B, Y112.B, G114.B, T115.B, G116.B, P117.B, A119.B, D144.B, H145.B, I146.B, G147.B, T148.B, R149.B, N150.B, N153.B, N154.B, A155.B, A156.B, I157.B, V158.B, L159.B, Q160.B
	4	Ninetedanib	RNA Binding Pore		[2.0]	Site finder. Overlaps with RNA binding residues (PDB ID: 7ACS and 7ACT). Residues: A50.A, S51.A, F53.A, T54.A, R88.A, Y109.A, Y111.A, P117.A, R149.A, A156.A, N153.B
	5 (CTD)	-	PPI	2CJR*[23]	[1.7]	Site finder. Residues: R15, Q16, K17, R18, T19, A20, V26, F30, R33, G34, P35, E36, Q37, T38, Q39, G40, N41, F42, G43, L47, G51, T52, W57, I60, A61, F63, A64, P65, S66, A67, F70, W86, L87, T88, Y89, T90, G91, A92, I93, L95, L109, I113

Table S2. Site Nomenclature and Information *Homology Model Template PDB ID

Table S3. 'Medium Confidence Hits' and Putative Mechanism of Hits with a z-score > 1.8 at fewer than 10 sites.

Structure/Entry			Processing			RT complex			Host interactions?		
Drug	Z-Score	Site	Drug	Z-Score	Site	Drug	Z-Score	Site	Drug	Z-Score	Site
Fedratinib	2.61	Spike 1	Aminolevulinic acid	2.86	NSP 3 PLP 3	**Eliglustat**	**3.03**	**NSP 7,8,12 3**	**Fedratinib**	**2.86**	**NSP 3 SUD**
**Amprenavir**	**2.47**	**Spike 6**	Eliglustat	2.44	NSP 5 3	Fedratinib	2.89	NSP10	Raloxifene	2.62	NSP15 3
Olmesartan	2.40	Spike 2	Fedratinib	2.34	NSP 5 3	Dipivefrin	2.60	NSP 7,8,12 3	**Domperidone**	**2.53**	**NSP 3 ADRP**
**Nintedanib**	**2.35**	**N 4**	Domperidone	2.30	NSP 3 PLP 4	Metoprolol	2.54	NSP 7,8,12 3	**Terconazole**	**2.52**	**NSP 3 ADRP**
Capsaicin	2.33	Spike 7	Avanafil	2.21	NSP 3 PLP 2	Remdesivir	2.53	NSP13	Avanafil	2.43	NSP15 4
**Oxybutynin**	**2.32**	**N 3**	Amprenavir	2.11	NSP 5 2	Fedratinib	2.42	NSP 7,8,12 1	Tamoxifen	2.28	NSP 3a
**Nintedanib**	**2.26**	**E 1**	Amprenavir	2.11	NSP 5 1	Tamoxifen	2.35	NSP14 5	**Alvimopan**	**2.25**	**NSP 1**
Calcipotriol	2.21	Spike 8	Avanafil	1.98	NSP 5 3	Eliglustat	2.27	NSP14 SAM	Avanafil	2.24	NSP15 2
Remdesivir	2.21	Spike 8	Eliglustat	1.98	NSP 3 PLP 2	Fedratinib	2.09	NSP14 SAM	Amprenavir	2.23	NSP15 1
Fedratinib	2.13	E 2	Remdesivir	1.93	NSP 3 PLP 3	Remdesivir	2.01	NSP 7,8,12 4	Domperidone	2.16	NSP 3 SUD
Eliglustat	2.12	Spike 7	Terconazole	1.88	NSP 5 2	Remdesivir	1.99	NSP14 2	Amprenavir	2.09	NSP15 3
Levomefolic acid	2.06	Spike 1	Bosutinib	1.88	NSP 5 1	Remdesivir	1.99	NSP14 3	**Fedratinib**	**2.03**	**NSP 3 NAB**
Remdesivir	2.04	E 2	Alvimopan	1.82	NSP 5 2	Fedratinib	1.98	NSP13	Alfuzosin	1.96	NSP 3 SUD
Ospemifene	2.03	N 3				Acebutolol	1.98	NSP 7,8,12 3	**Rofecoxib**	**1.93**	**NSP 4 2**
Oxybutynin	2.01	Spike 7				Eliglustat	1.98	NSP 7,8,12 5	Eliglustat	1.92	NSP15 1
Terconazole	2.01	Spike 2				Capsaicin	1.98	NSP 7,8,12 3	Remdesivir	1.92	NSP 3 NAB
Nintedanib	2.00	E 2				Vardenafil	1.96	NSP 7,8,12 2	Domperidone	1.85	NSP 3 NAB
Nintedanib	1.99	Spike 6				Bosutinib	1.95	NSP 7,8,12 2	Amiodarone	1.81	NSP15 1
Ivabradine	1.98	N 3				Eliglustat	1.93	NSP16	Lomitapide	1.80	NSP 4 2
Levomefolic acid	1.94	Spike 4				Amiodarone	1.91	NSP 7,8,12 1			
Cilostazol	1.94	Spike 7				Verapamil	1.91	NSP14 5			
Remdesivir	1.93	N 3				Tamoxifen	1.88	NSP14 SAM			
Avanafil	1.92	Spike 4				Capsaicin	1.86	NSP 9 2			
Cilostazol	1.88	Spike 8				Eliglustat	1.85	NSP14 2			
Dasatinib	1.88	N 3				Eliglustat	1.85	NSP14 3			
Eliglustat	1.87	E 2				Ospemifene	1.83	NSP14 5			
Remdesivir	1.86	N 2				Domperidone	1.83	NSP10			
Clofazimine	1.86	E 1				Hydroxychloroquine	1.80	NSP 9 1			
Aminolevulinic acid	1.86	Spike 3				Amprenavir	1.80	NSP14 2			
Fedratinib	1.84	Spike 4				Amprenavir	1.80	NSP14 3			
Acebutolol	1.83	Spike 2				Aminolevulinic acid	1.80	NSP16			
Clofazimine	1.83	E 2									
Tamoxifen	1.82	N 3									
Alvimopan	1.80	N 5									

Table S4. List of 105 hits from the *in vitro* assay against SARS-CoV-2 infection in Huh-7 cells with potency ≤ 2 μM with methods as described in [10].

Drug Name	DrugBank ID	Drug Name	DrugBank ID	Drug Name	DrugBank ID
Acebutolol	DB01193	Ethionamide	DB00609	Nintedanib	DB09079
Acetazolamide	DB00819	Ethosuximide	DB00593	Olmesartan	DB00275
Acetohexamide	DB00414	Ethotoin	DB00754	Omeprazole	DB00338
Alfuzosin	DB00346	Ezetimibe	DB00973	Ondansetron	DB00904
Allopurinol	DB00437	Fedratinib	DB12500	Ospemifene	DB04938
Alvimopan	DB06274	Fludarabine	DB01073	Oxybutynin	DB01062
Aminolevulinic acid	DB00855	Fludrocortisone	DB00687	Pamidronic acid	DB00282
Amiodarone	DB01118	Gemcitabine	DB00441	Phentolamine	DB00692
Amprenavir	DB00701	Gilteritinib	DB12141	Phenytoin	DB00252
Apraclonidine	DB00964	Glasdegib	DB11978	Procainamide	DB01035
Avanafil	DB06237	Guanethidine	DB01170	Pyridoxine	DB00165
Bosutinib	DB06616	Hydroxychloroquine	DB01611	Quinestrol	DB04575
Calcipotriol	DB02300	Ingenol mebutate	DB05013	Raloxifene	DB00481
Capsaicin	DB06774	Ivabradine	DB09083	Remdesivir	DB14761
Carbinoxamine	DB00748	Ketorolac	DB00465	Rivastigmine	DB00989
Carmustine	DB00262	Lapatinib	DB01259	Rocuronium	DB00728
Cefotaxime	DB00493	Levamisole	DB00848	Rofecoxib	DB00533
Cefoxitin	DB01331	Levofloxacin	DB01137	Sevoflurane	DB01236
Chloroxine	DB01243	Levomefolic acid	DB11256	Sildenafil	DB00203
Chlorthalidone	DB00310	Lomitapide	DB08827	Simvastatin	DB00641
Cidofovir	DB00369	Loperamide	DB00836	Tafamidis	DB11644
Cilostazol	DB01166	Loratadine	DB00455	Tamoxifen	DB00675
Clofazimine	DB00845	Meclizine	DB00737	Terconazole	DB00251
Dasatinib	DB01254	Mepivacaine	DB00961	Tetrabenazine	DB04844
Dexmedetomidine	DB00633	Meropenem	DB00760	Thalidomide	DB01041
Dienogest	DB09123	Mestranol	DB01357	Thioridazine	DB00679
Dipivefrin	DB00449	Metformin	DB00331	Tioguanine	DB00352
Domperidone	DB01184	Metoclopramide	DB01233	Trimethadione	DB00347
Dorzolamide	DB00869	Metoprolol	DB00264	Ulipristal	DB08867
Duvelisib	DB11952	Naratriptan	DB00952	Valproic acid	DB00313
Edaravone	DB12243	Nebivolol	DB04861	Vardenafil	DB00862
Eflornithine	DB06243	Nevirapine	DB00238	Verapamil	DB00661
Eliglustat	DB09039	Niclosamide	DB06803	Vilazodone	DB06684
Entecavir	DB00442	Nifedipine	DB01115	Zanamivir	DB00558
Eslicarbazepine acetate	DB09119	Nilutamide	DB00665	Zonisamide	DB00909

Table S5. Reference drugs identified using FQ score.

Drug Name	DrugBank ID	Molecular Weight	Drug Type	Targets	Z Score
Alvimopan	DB06274	424.53	Peripherally acting μ opioid receptor antagonist	NSP1	2.25
Aminolevulinic Acid	DB00855	131.13	Photochemotherapy for actinic keratosis	NSP3-PLP, Site 3	2.86
Amprenavir	DB00701	505.63	HIV protease inhibitor	Spike Protein, Site 6	2.47
				NSP15, Site 1	2.23
				NSP5, Site 2	2.11
				NSP5, Site 1	2.11
				NSP15, Site 3	2.09
Avanafil	DB06237	484.95	Phosphodiesterase-5 inhibitor for erectile dysfunction	NSP15, Site 4	2.43
				NSP15, Site 2	2.24
				NSP3-PLP, Site 2	2.21
Calcipotriol	DB02300	412.60	Treatment of plaque psoriasis	Spike Protein, Site 8	2.21
Capsaicin	DB06774	305.41	Topical analgesic	Spike Protein, Site 7	2.33
Dipivefrin	DB00449	351.44	Prodrug of adrenaline	NSP7,8,12, Site 3	2.60
Domperidone	DB01184	425.91	Dopamine receptor antagonist	NSP3-ADRP	2.53
				NSP3-PLP, Site 4	2.30
				NSP3-SUD	2.16
Eliglustat	DB09039	404.55	Glucosylceramide synthase inhibitor	NSP7,8,12, Site 3	3.03
				NSP5, Site 3	2.44
				NSP14, Site 1	2.27

				Spike Protein, Site 7	2.12
Levomefolic Acid	DB11256	459.46	Food additive for folate deficiency	Spike Protein, Site 1	2.06
Metoprolol	DB00264	267.36	β1 adrenergic receptor antagonist	NSP7,8,12, Site 3	2.54
Nintedanib	DB09079	539.62	Kinase inhibitor	Nucleocapsid, Site 4	2.35
				Envelope Protein, Site 1	2.26
Olmesartan	DB00275	446.50	Angiotensin II receptor antagonist	Spike Protein, Site 2	2.40
Ospemifene	DB04938	378.89	Selective estrogen receptor modulator	Nucleocapsid, Site 3	2.03
Oxybutynin	DB01062	357.49	Relief of overactive bladder	Nucleocapsid, Site 3	2.32
				Spike Protein, Site 7	2.01
Tamoxifen	DB00675	371.51	Selective estrogen receptor modulator	NSP14, Site 5	2.35
				NSP3-NSP3a	2.28
Terconazole	DB00251	532.46	14-alpha-demethylase inhibitor in fungi	NSP3-ADRP	2.52
				NSP3-PLP, Site 1	2.11
				Spike Protein, Site 2	2.01

Table S6. Number of Ions used in MD Simulations.

**Protein**	**Drug**	**Na^+ Ions**	**Cl^- Ions**
NSP14	Tamoxifen	0	9
NSP15	Avanafil	162	0
NSP1	Alvimopan	6	0
NSP3-ADRP	Domperidone	0	0
NSP3-ADRP	Terconazole	0	0
NSP3-PLP	Aminolevulinic Acid	3	0
NSP7,8,12	Dipivefrin	12	0
NSP7,8,12	Eliglustat	12	0
NSP7,8,12	Metoprolol	12	0
Nucleocapsid	Ospemifene	0	84
Nucleocapsid	Oxybutynin	0	85
Nucleocapsid	Nintedanib	0	85
Spike	Levomefolic Acid	59	0
Spike	Olmesartan	59	0
Spike	Amprenavir	57	0
Spike	Capsaicin	57	0
Spike	Calcipotriol	57	0

3. Supplemental References

1. Almeida, M. S., Johnson, M. A., Herrmann, T., Geralt, M. & Wüthrich, K. Novel beta-barrel fold in the nuclear magnetic resonance structure of the replicase nonstructural protein 1 from the severe acute respiratory syndrome coronavirus. *J Virol* **81**, 3151-3161 (2007).

2. Serrano, P. *et al.* Nuclear magnetic resonance structure of the N-terminal domain of nonstructural protein 3 from the severe acute respiratory syndrome coronavirus. *J Virol* **81**, 12049-12060 (2007).

3. Michalska, K. *et al.* Crystal structures of SARS-CoV-2 ADP-ribose phosphatase: from the apo form to ligand complexes. *IUCrJ* **7**, 814-824 (2020).

4. Tan, J. *et al.* The SARS-unique domain (SUD) of SARS coronavirus contains two macrodomains that bind G-quadruplexes. *PLoS Pathog* **5**, e1000428, doi:10.1371/journal.ppat.1000428 (2009).

5. Osipiuk, J. *et al.* The crystal structure of papain-like protease of SARS CoV-2. Nature Communications, **12**, 743 (2021).

6. Zhang, L. *et al.* Crystal structure of SARS-CoV-2 main protease provides a basis for design of improved α-ketoamide inhibitors. *Science* **368**, 409-412 (2020).

7. Serrano, P. *et al.* Nuclear magnetic resonance structure of the nucleic acid-binding domain of severe acute respiratory syndrome coronavirus nonstructural protein 3. *J Virol* **83**, 12998-13008 (2009).

8. Xu, X., Z. Lou, Y. Ma, X. Chen, Z. Yang, X. Tong, Q. Zhao, Y. Xu, H. Deng, M. Bartlam and Z. Rao. "Crystal structure of the C-terminal cytoplasmic domain of non-structural protein 4 from mouse hepatitis virus A59." PLoS One **4**(7): e6217 (2009).

9. Ghosh, A. K. *et al.* Severe acute respiratory syndrome coronavirus papain-like novel protease inhibitors: design, synthesis, protein-ligand X-ray structure and biological evaluation. *J Med Chem* **53**, 4968-4979 (2010).

10. Báez-Santos, Y. M. *et al.* X-ray structural and biological evaluation of a series of potent and highly selective inhibitors of human coronavirus papain-like proteases. *J Med Chem* **57**, 2393-2412 (2014).

11. Owen, C. D. *et al.* SARS-CoV-2 main protease with unliganded active site. PDB ID 6YB7, DOI: 10.2210/pdb6YB7/pdb (2019).

12. Douangamath, A. *et al.* Crystallographic and electrophilic fragment screening of the SARS-CoV-2 main protease. *Nat Commun* **11**, 5047 (2020).

13. Gao, Y. *et al.* Structure of the RNA-dependent RNA polymerase from COVID-19 virus. *Science* **368**, 779-782 (2020).

14. Tan, K., Kim, Y., Jedrzejczak, R., Maltseva, N., Endres, M., Michalska, K., Joachimiak, A., The crystal structure of Nsp9 RNA binding protein of SARS CoV-2, PDB ID 6W4B, DOI: 10.2210/pdb6W4B/pdb (2020).

15. Rosas-Lemus, M. *et al.* High-resolution structures of the SARS-CoV-2 2'-. *Sci Signal* **13**, 651 (2020).

16. Jia, Z. *et al.* Delicate structural coordination of the Severe Acute Respiratory Syndrome coronavirus Nsp13 upon ATP hydrolysis. *Nucleic Acids Res* **47**, 6538-6550 (2019).

17. Ma, Y. *et al.* Structural basis and functional analysis of the SARS coronavirus nsp14-nsp10 complex. *Proc Natl Acad Sci U S A* **112**, 9436-9441 (2015).

18. Kim, Y. *et al.* Crystal structure of Nsp15 endoribonuclease NendoU from SARS-CoV-2. *Protein Sci* **29**, 1596-1605 (2020).

19. Walls, A. C. *et al.* Structure, Function, and Antigenicity of the SARS-CoV-2 Spike Glycoprotein. *Cell* **181**, 281-292 (2020).

20. Shang, J. *et al.* Cell entry mechanisms of SARS-CoV-2. *Proc Natl Acad Sci U S A* **117**, 11727-11734 (2020).

21. Surya, W., Li, Y. & Torres, J. Structural model of the SARS coronavirus E channel in LMPG micelles. *Biochim Biophys Acta Biomembr* **1860**, 1309-1317 (2018).

22. Chang, C., Michalska, K., Jedrzejczak, R., Maltseva, N., Endres, M., Godzik, A., Kim, Y., Joachimiak, A. Center for Structural Genomics of Infectious Diseases (CSGID). PDB ID 6VYO, DOI: 10.2210/pdb6VYO/pdb (2020).

23. Chen, C. Y. *et al.* Structure of the SARS coronavirus nucleocapsid protein RNA-binding dimerization domain suggests a mechanism for helical packaging of viral RNA. *J Mol Biol* **368**, 1075-1086 (2007).
